# Nuclear phosphatidylinositol-5-phosphate regulates ING2 stability at discrete chromatin targets in response to DNA damage

**DOI:** 10.1038/srep02137

**Published:** 2013-07-04

**Authors:** Dennis J. Bua, Gloria Mas Martin, Olivier Binda, Or Gozani

**Affiliations:** 1 Department of Biology, Stanford University, Stanford, CA 94305, USA; 2 Current address: Northern Institute for Cancer Research, Newcastle University, Newcastle upon Tyne, United Kingdom.

## Abstract

ING2 (inhibitor of growth family member 2) is a component of a chromatin-regulatory complex that represses gene expression and is implicated in cellular processes that promote tumor suppression. However, few direct genomic targets of ING2 have been identified and the mechanism(s) by which ING2 selectively regulates genes remains unknown. Here we provide evidence that direct association of ING2 with the nuclear phosphoinositide phosphatidylinositol-5-phosphate (PtdIns(5)P) regulates a subset of ING2 targets in response to DNA damage. At these target genes, the binding event between ING2 and PtdIns(5)P is required for ING2 promoter occupancy and ING2-associated gene repression. Moreover, depletion of PtdIns(5)P attenuates ING2-mediated regulation of these targets in the presence of DNA damage. Taken together, these findings support a model in which PtdIns(5)P functions as a sub-nuclear trafficking factor that stabilizes ING2 at discrete genomic sites.

Chromatin structure plays a central role in the establishment, maintenance, and propagation of nuclear programs and the dysregulation of gene expression programs occurs in numerous human pathologies, including cancer. A key mechanism regulating chromatin structure involves the reversible covalent post-translational modification (PTM) of histone proteins by chemical moieties such as acetyl- and methyl-groups. Histone PTMs are dynamic, frequently changing in response to external cues^1^,^2^,^3^. For example, histone PTMs acutely change during DNA damage responses (DDRs)^4^,^5^,^6^. A key question in understanding how nuclear programs, such as DDRs, are appropriately orchestrated is to identify how chromatin-regulatory activities are specifically targeted and maintained at discrete chromosomal sites.

Inhibitor of Growth family member 2 (ING2) is a tumor suppressor protein^7^ that is a core component of a multi-factor chromatin-modifying complex containing the transcriptional co-repressor SIN3A and HDAC1 (histone deacetylase 1)^8^. In response to DNA damage, ING2 associates with chromatin where it modulates crosstalk between lysine methylation and lysine acetylation on histone proteins^9^. ING2 is stabilized at active chromatin via its PHD (Plant homeodomain) finger^9^,^10^, which selectively binds to H3K4me3 (Histone H3 trimethylated on lysine 4), a hallmark of active transcription^11^,^12^. Genomic profiling of H3K4me3 patterns in a variety of cell types conclude that H3K4me3 levels are high in the 5′ region of nearly all actively expressed genes and, consistently, H3K4me3 promoter enrichment correlates with active RNA polymerase occupancy, and histone hyper-acetylation^13^. Thus, the interaction between ING2 and H3K4me3 (ING2::K4me3) stabilizes ING2-SIN3A-HDAC1 complexes at target gene promoters, regions rich in acetylated histones, resulting in histone deacetylation and gene repression^9^,^14^. However, the mechanism(s) that senses DNA damage and places ING2 in the vicinity of H3K4me3-rich target gene promoters is not known.

Phospholipids, including phosphoinositides (PtdInsPs) play a pivotal role in cytoplasmic trafficking of intracellular proteins^15^,^16^. While relatively little is known about the roles of nuclear PtdInsPs compared to their cytosolic counterparts, recent evidence suggests that nuclear phosphoinositides control the localization of proteins in the nucleus^17^,^18^,^19^,^20^,^21^. The direct interaction between ING2 and nuclear phosphatidylinositol-5-phosphate (PtdIns(5)P) changes sub-nuclear distribution of ING2, which facilitates recruitment of ING2 to chromatin, and is required for ING2-associated activation of p53 during DDRs^17^,^22^,^23^. Interestingly, levels of PtdIns(5)P undergo dynamic fluctuations within the nucleus during DDRs. For example, etoposide, a topoisomerase inhibitor, increases the level of PtdIns(5)P in the chromatin of human fibrosarcoma cells (HT1080)^17^. These observations suggest a model in which dynamic changes in PtdIns(5)P levels could modulate selective chromatin targeting of ING2.

Here we demonstrate that the interaction between ING2 and nuclear PtdIns(5)P (ING2::PtdIns(5)P) directly regulates chromatin-mediated gene expression control. We first identify the DNA damage-associated gene expression network mediated by ING2 and then uncover the subset of these genes that are specifically modulated by association of ING2 with PtdIns(5)P. For this cluster, there is a loss of ING2-associated gene repression when ING2::PtdIns(5)P is disrupted or when nuclear PtdIns(5)P is metabolized. Loss of ING2 promoter occupancy is concomitant with abrogation of DNA damage-induced histone deacetylation resulting in aberrant transcript elevation. Taken together, these findings establish PtdIns(5)P as a new class of nuclear signaling molecules controlling gene expression and provide mechanistic insight into the role of sub-nuclear trafficking in the acute regulation of chromatin structure.

## Results

### Characterization of an ING2-associated DNA damage gene expression program

To test the role of PtdIns(5)P-binding by ING2 on ING2 gene expression functions, we determined the ING2-mediated DNA damage program using a comprehensive genomic approach coupling ChIP-chip (chromatin immunoprecipitation combined with tiling promoter DNA microarrays) and gene expression microarray analyses. Combining these assays allowed for the identification of gene promoters bound by ING2 as well as the downstream transcriptional changes. Once we established a baseline, we monitored changes to the ING2 expression network resulting from perturbations in PtdIns(5)P signaling (diagrammed in Figure S1).

**Figure 1:**
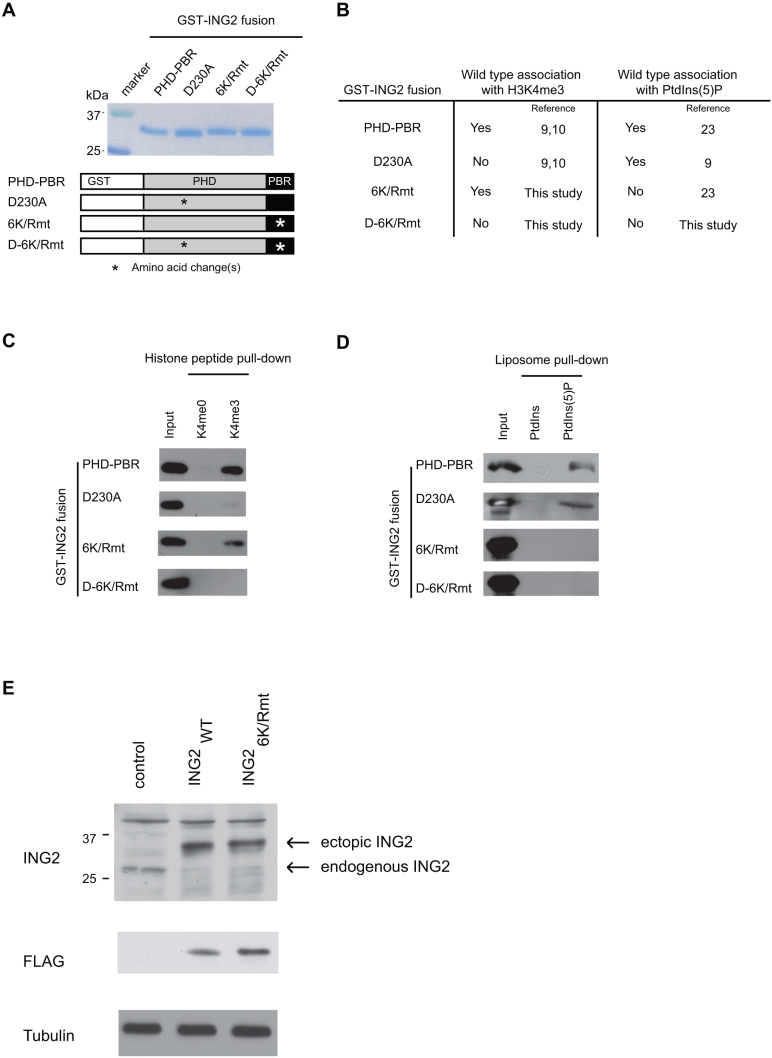
ING2 independently interacts with H3K4me3 and PtdIns(5)P. (A) (Top) Coomassie stain of GST-fused ING2 C-terminus constructs.(Bottom) Schematic showing protein domain organization in the ING2 constructs. Asterisk indicates site of mutagenesis in which the endogenous amino acid is replaced with alanine: in the PHD domain D230A; in the PBR domain K272A, K274A, K275A, R277A, R278A, and R280A. (B) Table summarizing GST-ING2 fusion binding activities. (C) Biotinylated histone pull-downs for ING2 constructs. (D) Liposome pull-downs for ING2 constructs. (E) Immunoblot panel of HT1080 cell systems constitutively expressing ectopic ING2 forms. Uncropped images of blots are shown in Supp Figure 3.

For this study it was necessary to identify a form of ING2 that does not bind PtdIns(5)P, but is not deficient in H3K4me3 binding. The C-terminus of ING2 (Figure 1A) contains two conserved modules (1) a PHD finger and (2) a polybasic region (PBR); within these modules several key residues have been characterized for their role in histone or lipid binding (see Figure 1B). For example, a specific single amino acid change within the PHD finger (ING2_D230A_) abrogates ING2::H3K4me3 without ablation of ING2::PtdIns(5)P^9^. Here we tested if a previously identified^23^ mutant deficient in PtdIns(5)P binding (named ING2_6K/Rmt_) can bind H3K4me3. Additionally we generated and characterized a mutant with the combined amino acid changes in ING2_D230A_ and ING2_6K/Rmt_ named ING2_D-6K/Rmt_. We subjected the panel of ING2 mutants to *in vitro* histone peptide (Figure 1C) and liposome pull-down assays (Figure 1D). As shown in Figure 1C, ING2_6K/Rmt_ which fails to bind to PtdIns(5)P-containing liposomes, binds to H3K4me3 comparable to wild-type. In contrast, ING2_D-6K/Rmt_ is deficient in binding to both H3K4me3 and PtdIns(5)P (Figure 1C,D). Thus ING2_6K/Rmt_ and the other ING2 mutants can be used to isolate the impact of the ING2::PtdIns(5)P on ING2 gene-regulatory functions. Next we generated human fibrosarcoma cells (HT1080) stably-expressing epitope-tagged, full-length wild-type ING2 (ING2_WT_) or ING2_6K/Rmut_. Note that endogenous ING2 protein levels are depleted in response to ectopic expression of wild-type and mutant ING2, thus the majority of ING2 in the reconstituted cells is exogenous (Figure 1E; data not shown).

Next, genome-wide promoter occupancy of ING2_WT_ was determined. DNA that co-purified with FLAG-ING2_WT_ in vehicle-treated and etoposide-treated HT1080 cells was hybridized to Nimblegen whole genome human promoter arrays and peaks were detected using Nimblescan software. Significant ING2 peaks (see Methods) were identified at over 200 unique promoters in etoposide-treated cells, whereas no significant ING2 peaks were detected in vehicle-treated cells (Supplementary Table 1). We hereafter refer to the set of genes selectively bound by ING2 in the presence of etoposide as ING2_ET-BOUND_ targets.

**Figure 2:**
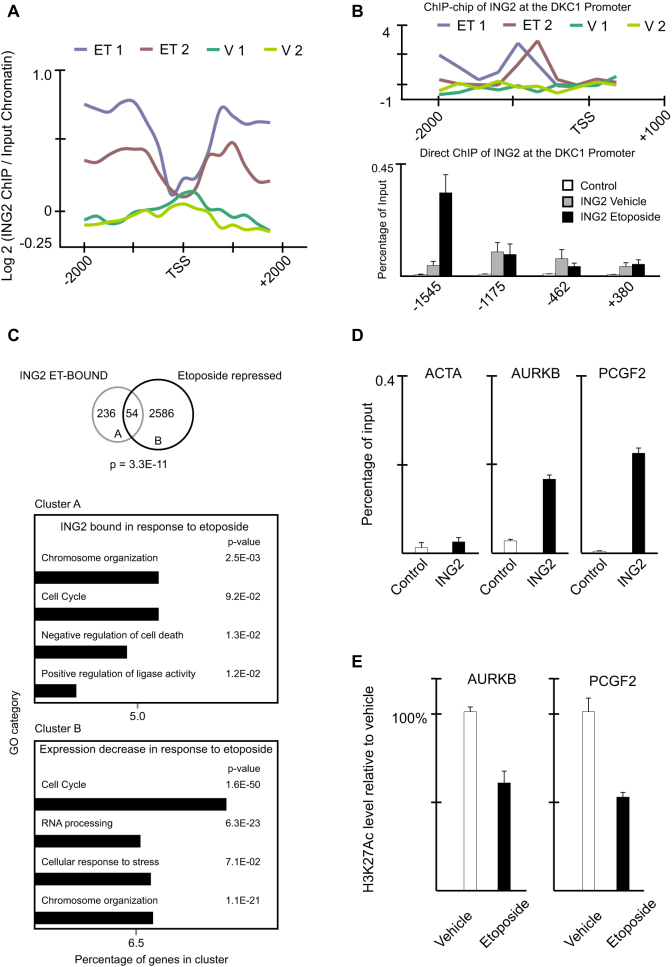
ING2 is a critical mediator of chromatin dynamics in the presence of DNA damage. (A) Dynamic association of ING2 with gene promoters in response to etoposide. Average ING2 promoter enrichment in ChIP-chip assays (two replicates of Etoposide (ET)-treated cells, two replicates of vehicle (V)-treated cells). (B) Direct ChIP of endogenous ING2 is consistent with FLAG-ING2 ChIP-chip determined promoter occupancy. (Top) ING2 enrichment at the DKC1 promoter in the indicated ChIP-chip experiments. (Bottom) Direct ChIP of endogenous ING2 at the promoter of DKC1 under conditions indicated. Control = no antibody control IP. (C) Enrichment of ING2_ET-BOUND_ genes in the set of transcripts repressed in response to DNA damage. (Top) Venn diagram highlighting the overlap between genes directly regulated by ING2 and genes repressed in response to etoposide. p-value indicates significance of overlap determined by Fisher's exact test. (Bottom) Gene Ontology (GO) analyses of ING2_ET-BOUND_ genes (Cluster A) or Etoposide-repressed genes (Cluster B). (D) ChIP of endogenous ING2 at the promoters of Actin (ACTA), Aurora Kinase B (AURKB), and Polycomb group RING finger protein 2 (PCGF2) in etoposide-treated cells. Control = no antibody control IP. (E) ChIP of Histone H3 acetylated at Lysine 27 (H3K27Ac) at the AURKB and PCGF2 promoters in the absence and presence of etoposide. See also Supp Figure 2 and Supp Table 1.

Heatmap displays and average binding profiles were generated to highlight ING2 occupancy within the promoters of ING2_ET-BOUND_ genes (Figure 2A; Figure S2). ING2 is enriched in promoters both upstream and downstream of the transcriptional start site (TSS) with decreased occupancy in the nucleosome-poor TSS, this is consistent with the known distribution of H3K4me3 dictating ING2 occupancy via the ING2::H3K4me3 interaction^9^,^12^,^24^. Next we tested if endogenous ING2 behaves the same as FLAG-tagged ING2 at a novel ING2 target we identified: *DKC1* (Figure 2B). The FLAG-ING2 ChIP-chip data (Figure 2B top) is consistent with direct ChIP assays of endogenous ING2 (Figure 2B bottom): ING2 associates with the *DKC1* promoter in the presence of etoposide with strong enrichment upstream of the TSS. Collectively, we identified a cluster of over two hundred novel gene promoters that ING2 selectively associates with in the presence of DNA damage.

To determine if etoposide-induced ING2 occupancy results in a functional change in transcription at ING2 target genes we performed a series of gene expression microarray experiments. Note that promoter occupancy was assayed 1 hr after etoposide treatment, while gene expression analyses were monitored 20 hrs after etoposide treatment to allow for transcriptional responses to occur. Global transcript profiling indicated that ING2_ET-BOUND_ genes (Cluster A) represent a statistically-significant (p = 3.3E-11, Fisher's exact test) population of genes repressed in response to etoposide (Cluster B; Figure 2C, top). Consistently, both of these gene clusters contain enrichment of factors that modulate chromosome organization and the cell cycle (Figure 2C, bottom). To further explore the relationship between ING2 promoter occupancy and transcriptional repression we tested more promoter targets. Endogenous ING2 is promoter-bound at *AURKB* (a representative from the cell cycle cluster) and *PCGF2* (a representative from the chromosome organization cluster), but not at *ACTA*, a negative control (Figure 2D). ING2 occupancy correlates with a decrease in acetylated H3 at lysine 27 (H3K27Ac; Figure 2E). These data are consistent with ING2 being present in an HDAC1 complex^8^,^9^,^14^. Taken together, these findings support the notion that ING2 plays a key role in DNA damage-induced gene repression, and this activity may be critical for ING2 tumor suppressive functions.

### The ING2-PtdIns(5)P interaction is required for execution of the ING2-associated DNA damage gene expression program

**Figure 3:**
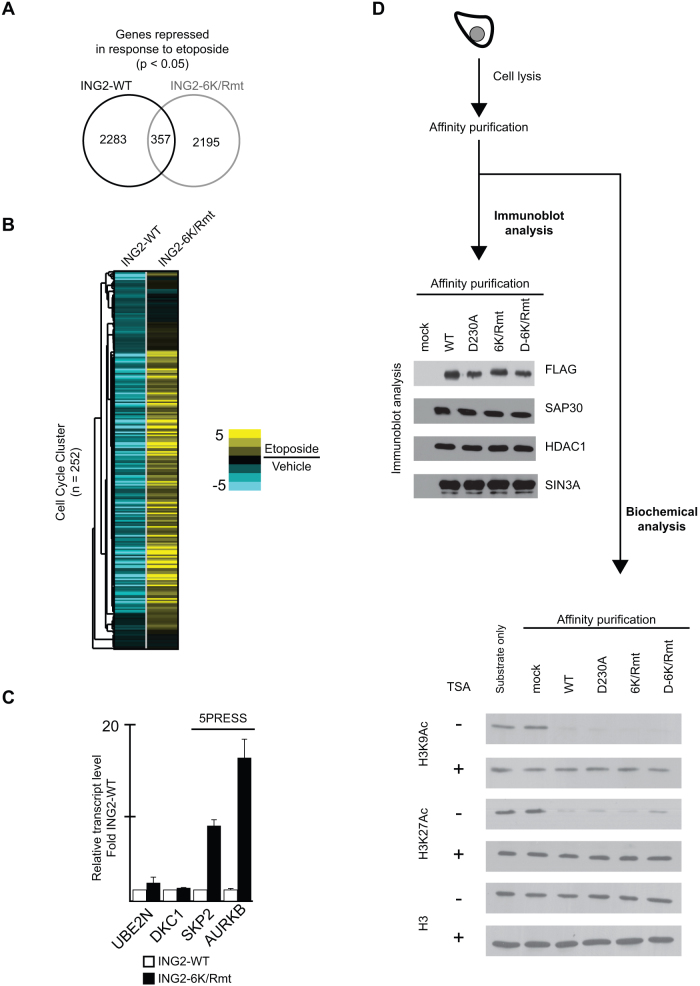
The ING2::PtdIns(5)P interaction is a critical modulator of the nuclear program mediated by ING2. (A) Reprogramming of transcriptional repression in cells lacking ING2-PtdIns(5)P. Venn diagram highlighting the overlap between genes repressed in response to etoposide in cells expressing ING2_WT_ or ING2_6K/Rmt_. (B) Massive impairment of Cell Cycle components when the ING2-PtdIns(5)P interaction is ablated. Comparison of gene expression changes of Cell Cycle modulators (Cluster B in Figure 2C) in cells expressing ING2_WT_ or ING2_6K/Rmt_. (C) The transcript level of 5PRESS ING2 targets (AURKB and SKP2) are elevated in cells lacking the ING2::PtdIns(5)P interaction. The mRNA levels (determined by qPCR) of ING2 Etoposide-bound targets: AURKB, DKC1, SKP2, and UBE2N. (D) Full-length ING2_6K/Rmt_ purified from cells associates with comparable HDAC activity as ING2_WT_. (Top) Immunoblot analysis of FLAG immunoprecipitates from HT1080 populations stably-expressing: ING2_WT_, ING2_D230A_, ING2_6K/Rmt_, ING2_D-6K/Rmt_, or empty vector (control). (Bottom) Immunoblot analysis of *in vitro* HDAC reactions on bulk, purified histones. Where indicated, reactions were performed in the presence of trichostatin A (TSA). Uncropped images of blots are shown in Supp Figure 4.

Based on the observations that (1) ING2 dynamically associates with chromatin in response to etoposide to repress transcription, and (2) nuclear PtdIns(5)P levels increase in response to etoposide-treatment, we postulated that DDR-induced ING2-PtdIns(5)P interactions would regulate ING2 occupancy and/or activity at target genes. To test this we performed global gene expression analyses on cells expressing ING2_6K/Rmt_. Transcript profiling indicates that ablation of ING2::PtdIns(5)P results in impairment of etoposide-induced nuclear programs (Figure 3). Since ING2 is involved in gene repression, we focused on down-regulated transcripts. Few down-regulated transcripts overlap (<15%) when comparing cells expressing ING2_WT_ to ING2_6K/Rmt_, suggesting an unraveling of the appropriate DNA-damage induced transcription response in cells in which ING2 is unable to bind PtdIns(5)P (Figure 3A). Heatmap analysis of cell cycle regulators (genes that comprise Cluster B from Figure 1C) uncovered a striking expression signature: loss of etoposide-induced repression in cells expressing the ING2 lipid-binding mutant (Figure 3B); more specifically, genes that are strongly repressed in response to etoposide in ING2_WT_ cells are aberrantly activated in ING2_6K/Rmt_ cells. Hereafter this expression pattern is called 5PRESS (transcripts that require the binding event between ING2 and PtdIns(5)P for repression in response to etoposide). Quantitative PCR transcript (qPCR) analyses were performed on several ING2_ET-BOUND_ targets to confirm the 5PRESS expression signature (Figure 3C). In cells treated with etoposide the transcript level of *UBE2N* and *DKC1* are similar in cells ectopically expressing ING2_WT_ or ING2_6K/Rmt_. In contrast, the transcript levels of *SKP2* and *AURKB* (5PRESS targets) are elevated over 5-fold in cells expressing ING2_6K/Rmt_ compared to cells expressing ING2_WT_. To summarize, transcript analyses indicate that inhibition of the ING2-PtdIns(5)P interaction results in aberrant DDR-induced gene repression.

One possible explanation for the defect in gene repression of cells expressing ING2_6K/Rmt_ is that this mutant has decreased association with HDAC activity. To investigate this possibility, a panel of ING2 complexes were purified then tested for *in vitro* HDAC activity (Figure 3D). Compared to ING2_WT_, affinity-purified ING2_6K/Rmt_ associated with comparable levels of HDAC1, SAP30, and SIN3A (Figure 3D, top). Furthermore, affinity-purified ING2_6K/Rmt_ exhibits *in vitro* histone deacetylase (HDAC) activity (on H3K9 and H3K27) comparable to ING2_WT_ (Figure 3D, bottom). We conclude that ablation of ING2::PtdIns(5)P does not alter ING2-associated HDAC activity. Taken together, these results strongly argue a direct role for PtdIns(5)P in mammalian gene regulation.

### PtdIns(5)P is required for stabilization at a spectrum of ING2 promoter targets

**Figure 4:**
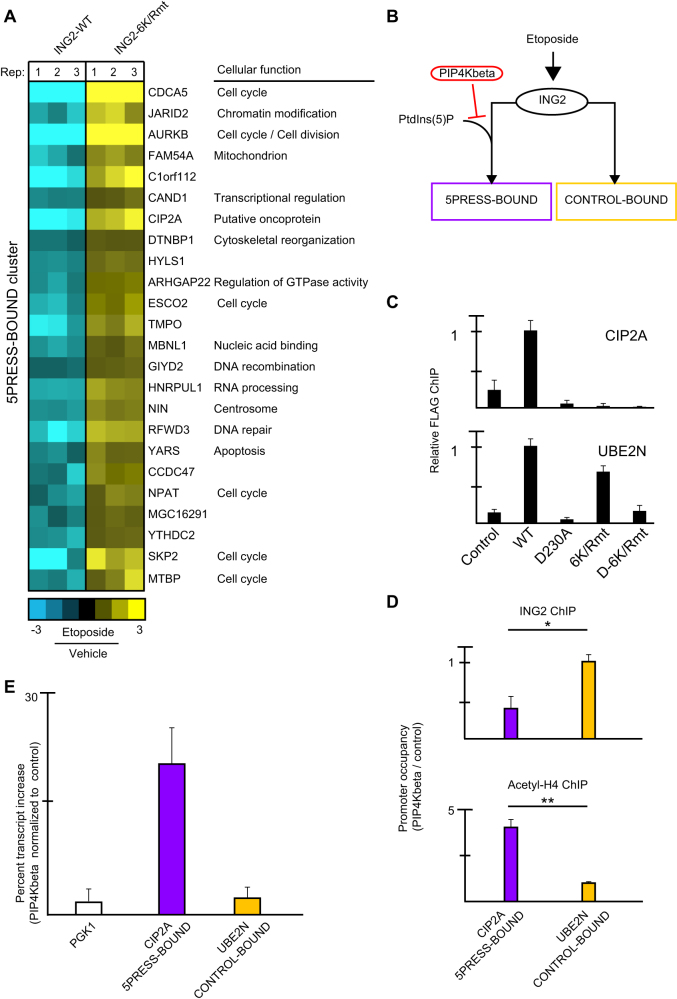
Select ING2 targets require PtdIns(5)P for promoter stabilization. (A) ING2 direct targets that require ING2-PtdIns(5)P for DNA damage-induced repression (5PRESS-BOUND) are involved in diverse processes. Rep = Biological replicate. (B) Schematic of model in which ING2_ET-BOUND_ genes are divided into two groups. Predicted effect of PIP4Kbeta over-expression on ING2 target gene regulation is highlighted. (C) ING2_6K/Rmt_ is promoter-bound at UBE2N, but not promoter-bound at CIP2A. Relative anti-FLAG ING2 ChIP from HT1080 cell lines above treated with etoposide (100 uM) for 1 hr. (D) Over-expression of PIP4Kbeta results in decreased ING2 occupancy as well as increased acetylation of histone H4 at the 5PRESS-BOUND representative, CIP2A. Direct ChIP was performed with indicated antibodies in cells treated with etoposide. Data represent averages of 3 independent experiments. * p = 4.86E-02, ** p = 4.43E-02, one-tailed Student's *t*-test. (E) Ectopic expression of PIP4Kbeta impairs ING2-mediated repression in the presence of etoposide. mRNA transcript levels of CIP2A are aberrantly elevated with little effect on UBE2N or PGK1 in cells expressing PIP4Kbeta. Data represent averages of 3 independent experiments.

To focus functionally on the role of PtdIns(5)P in selective gene repression we narrowed our attention to genes directly bound by ING2 that require ING2::PtdIns(5)P for repression (hereafter called ING2_5PRESS-BOUND_; Figure 4A). More specifically, ING2_5PRESS-BOUND_ targets: (1) are ING2_ET-BOUND_, indicating that ING2 is selectively promoter-bound in the presence of etoposide (Supp Figure 2, Supp Table 1); (2) have a significant transcript change determined by ANOVA (analysis of variance), which accounts for inter- and intra-group variability of all the microarrays used in this study; and (3) have the 5PRESS expression signature. PtdIns(5)P modulates repression of ~10% of ING2 direct targets, note the remaining ING2 targets are hereafter referred to as ING2_CONTROL-BOUND_ (Figure 4A).This group of genes is involved in diverse cellular processes such as cell cycle control, organelle homeostasis, and RNA processing (Figure 4A). Intriguingly, the ING2-PtdIns(5)P interaction is required for repression of *CIP2A* (also known as *KIAA1524*), a putative oncogene that promotes anchorage-independent growth^25^.

Our working model was that select ING2 targets require stable association of ING2 with PtdIns(5)P to properly traffic ING2 to the promoter (Figure 4B). To test our model we determined if ablation of ING2::PtdIns(5)P would result in loss of ING2 promoter targeting. Promoter occupancy for the ING2 panel was tested at an ING2_5PRESS-BOUND_ representative, *CIP2A*, as well as an ING2_CONTROL-BOUND_ representative, *UBE2N*, in which ING2 promoter occupancy should not be regulated by PtdIns(5)P (Figure 4C). ING2_6K/Rmt_ associates with the promoter of *UBE2N*, but is not promoter-bound at *CIP2A*. Furthermore, neither ING2_D230A_ nor ING2_D-6K/*Rmt*_ are enriched compared to the control IP (FLAG-IP from mock-transduced cells), which is consistent with the critical role of H3K4me3::ING2 interaction in stabilizing ING2 at target promoters. These data support the hypothesis that ING2::PtdIns(5)P is required for promoter occupancy at discrete ING2 chromatin targets.

Next we determined if chromatin trafficking of endogenous ING2 is impaired in an established cell-based system in which PtdIns(5)P signaling is depleted due to over-expression of the human enzyme phosphatidylinostiol-4-phosphate kinase II beta (PIP4Kbeta), which utilizes PtdIns(5)P as a substrate to generate PtdIns(4,5)P_2_^17^,^26^. PIP4Kbeta expression abrogates association of endogenous ING2 with the ING2_5PRESS-BOUND_ representative, *CIP2A*, but not with the ING2_CONTROL-BOUND_ representative, *UBE2N* (Figure 4D top). Furthermore in PIP4Kbeta-expressing HT1080 cells, acetylated histone H4 (Acetyl-H4) levels are elevated exclusively at the *CIP2A* promoter (Figure 4D bottom). These promoter changes have a functional consequence on gene expression since ectopic PIP4Kbeta results in a loss of DDR-induced gene repression for *CIP2A*, but not *UBE2N* or *PGK1*, a gene not directly regulated by ING2 (Figure 4E). Thus, disrupting the interaction between ING2 and PtdIns(5)P by two independent methods impairs the ability of ING2 to bind and subsequently repress transcription of a subset of ING2 target genes.

## Discussion

An understanding of the gene network controlled by ING2 is critical to unravel the molecular mechanism by which ING2 functions under physiological conditions as a tumor suppressor protein. Here, we uncovered several key features of the nuclear program mediated by ING2 in response to DNA damage. We identified more than 200 novel gene promoters directly bound by ING2 in response to genotoxic stress. Consistent with tumor suppressive functions, ING2 regulates several key cellular processes including chromatin structure, cell cycle progression, and cell proliferation. We focused on a group of ING2 targets that are dependent on the nuclear lipid PtdIns(5)P for proper regulation. The genes controlled by PtdIns(5)P are dysregulated when either (1) the binding event between ING2 and PtdIns(5)P is abrogated or (2) PtdIns(5)P levels are depleted due to PIP4Kbeta over-expression. Together, our data suggest that PtdIns(5)P acts to localize ING2 to target gene promoters which is instrumental in focusing ING2-associated HDAC activity. PtdIns(5)P was discovered nearly two decades ago^27^, and our work provides a new molecular mechanism of how PtdIns(5)P can function in the context of signal transduction within the nucleus.

Chromatin and phospholipid signaling networks impact gene expression and dysregulation of either one of these networks can contribute to human pathological states including cancer. ING2 is of great interest because it is a direct integrator of chromatin and nuclear phosphoinositide signaling networks and here we report that these pathways converge to regulate the putative oncogene *CIP2A*. Our data favor a model in which the ING2::PtdIns(5)P interaction stabilizes ING2 at discrete sites within the nucleus, where ING2 can then bind to H3K4me3 and anchor ING2-HDAC1-SIN3 complexes at gene promoters. While this study identifies a specialized role for ING2::PtdIns(5)P in gene expression control, it also highlights that ING2::H3K4me3 may have a more general role in gene regulatory functions. In Figure 4C we find that the mutant of ING2 unable to bind H3K4me3 is not bound at either ING2 direct target. While histone PTMs clearly have important roles in the stabilization and maintenance of regulatory complexes on the chromatin template, selective control of gene expression requires a large repertoire of factors including DNA sequence-specific factors and other regulatory cofactors^28^. Here we present evidence that nuclear phosphoinositides may participate as regulatory cofactors playing a critical role in chromatin dynamics. In contrast to the numerous chromatin-binding domains that have been characterized, few predominantly nuclear lipid-binding proteins have been characterized. However, a recent report elaborately employed a proteomic approach to identify over one hundred nuclear proteins that interact with the phosphoinositide PtdIns(4,5)P_2_^29^. We speculate that follow-up studies on some of these candidates, as well as discovery of additional PtdInsP-effectors, will uncover the diversity of functions of nuclear PtdInsPs in gene regulation and other fundamental nuclear processes.

## Methods

### Histone-peptide and liposome binding assays

Biotinylated H3 peptides and histone-peptide binding assays have been described previously^9^. Lipids were purchased from Echelon biosciences or Avanti Polar lipid. Liposome binding assays were carried out essentially as described in^30^. Briefly, recombinant protein and liposomes were combined in binding buffer (50 mM Tris, pH 7.5, 150 mM NaCl, 0.05% Nonidet P-40), rotated for 1 hr at room temperature, and then centrifuged at 13,000 rpm for 2 min. The liposome pellet was washed three times in 1 ml of binding buffer. Liposome pellets were resolved by SDS-PAGE, and GST-fusion proteins were detected by Immunoblot using an anti-GST antibody (Abcam).

### ChIP and ChIP-chip

HT1080 cells were treated with Etoposide (100 uM) or vehicle (DMSO) for 1 hr, then prepared as described previously for chromatin immunoprecipitation (ChIP)^31^. Purified ChIP DNA was quantified by real-time PCR (RT-PCR) using a LightCycler 480 II (Roche) or a 7300 Real Time PCR machine (Applied Biosystems). Primers are listed in Supp Table 3. For direct ChIP experiments (unless otherwise indicated), a representative experiment is shown with error bars indicating standard error of the mean (s.e.m.) of three technical replicates. ChIP-chip assays were performed as previously^31^. Briefly, ChIP DNA purified as described above, then amplified (Sigma Whole Genome Amplification kits 2 and 3), labeled, and hybridized to Roche Nimblegen 385 K RefSeq promoter arrays. ChIP-chip peaks were identified using the Nimblescan algorithm according to the standard manufacturer's protocol. A peak must have a minimum of four consecutive ChIP-chip probes where the log2 ratio of ChIP to input signal is between 20-90% of the hypothetical maximum log2 ratio (the mean log2 ratio of the array + 6 standard deviations). The false discovery rate (FDR) for each peak was calculated by permutations of the data within each chromosome, our cutoff was FDR less than or equal to 0.2. We applied an additional cutoff which required the fourth highest probe within the peak to have a log2 ratio > 0.5 (ING2 ChIP/Input chromatin). To be a significant ING2 peak, the gene had to meet all of the criteria above in both biological replicates.

### Antibodies and Plasmids

ING2 and derivative constructs were previously described^9^,^23^. PIP4Kbeta and derivative constructs were previously described or sub-cloned into: pBabe with FLAG-HA epitopes^9^,^23^. Mutants were made by PCR-mediated site-directed mutagenesis (Stratagene). Antibodies used in this study: ING2^23^, H3K4me3 (Active Motif); SAP30, HDAC1 and Acetyl-H4 (Millipore); Tubulin (Upstate); H3, H3K27ac, HDAC1 and SIN3A (Abcam); Rabbit IgG, FLAG M2 and H3K9ac (Sigma).

### Transcript analyses

RNA was isolated using RNeasy plus kits (Qiagen). For gene expression microarrays, RNA was processed for use with Illumina Bead Arrays per manufacturer's instructions. Probe-level data were processed using Genespring software and all subsequent analyses were performed using MeV^32^,^33^. Gene Ontology (GO) analysis was performed using the DAVID Bioinformatics Resource^34^,^35^. For quantitative transcript analysis by qPCR, RNA was isolated as above and then converted to DNA using the Superscript III first-strand synthesis system (Invitrogen). Primer pairs and Roche Universal Probe information are listed in Supplementary Table 3. The comparative threshold method was used for relative quantification and each gene was normalized to GAPDH. Unless otherwise stated, a representative experiment is presented with error bars indicating s.e.m. of three technical replicates.

### Cell culture, transient transfection, viral transduction, enzymatic assays

293T and HT1080 cell lines (ATCC) were cultured in Advanced DMEM (Invitrogen) supplemented with penicillin-streptomycin (Invitrogen), glutamine (Invitrogen), and 10% newborn calf serum (Atlanta). Transient transfection, retroviral transduction, and histone deacetylation (HDAC) reactions were performed as previously described^9^. For HDAC reactions, full-length FLAG-ING2 complexes were purified from mammalian cells lysed in buffer (50 mM Tris-HCl (pH7.4), 250 mM NaCl, 0.5% Triton X-100, 10% glycerol, 1 mM PMSF, Protease Inhibitors (Roche)) using anti-FLAG M2-conjugated agarose (Sigma) and eluted with 0.4 mg/ml FLAG peptide. The substrate used was calf thymus histones (Worthington) and where indicated the reactions were performed in the presence of 330 nM trichostatin A (Sigma).

## Author Contributions

Conceived and designed the experiments: D.J.B., G.M., O.B., O.G. Performed the experiments: D.J.B., G.M., O.B. Analyzed the data: D.J.B., G.M., O.B., O.G. Wrote the main manuscript text: D.J.B., O.G. Reviewed the manuscript: D.J.B., G.M., O.B., O.G.

## Supplementary Material

Supplementary Information Supplementary Info File #1
