# Supplementary material for: Nuclear phosphatidylinositol-5-phosphate regulates ING2 stability at discrete chromatin targets in response to DNA damage
Source: Sci Rep. 2013 Jul 4;3:2137. doi: 10.1038/srep02137 (PMC3705588; doi:10.1038/srep02137)
Supplement: Supplementary Information — Supplementary Info File #1 [file srep02137-s1.pdf]

Supplementary Information for:

Nuclear phosphatidylinositol-5-phosphate regulates ING2 stability at discrete chromatin targets in response to DNA damage

Dennis J. Bua[1], Gloria Mas Martin[1], Olivier Binda[1,#], Or Gozani[1,*]

[1]*Department of Biology, Stanford University, Stanford, CA 94305, USA*

[*]To whom correspondence should be addressed: ogozani@stanford.edu

[#]Current address: Northern Institute for Cancer Research, Newcastle University, Newcastle upon Tyne, United Kingdom

Bua et al. Supplementary Information

Contents

Supplementary Figure 1
Supplementary Figure 2
Supplementary Figure 3
Supplementary Figure 4
Supplementary Table 1
Supplementary Table 2

Supplementary Figure 1: Workflow to identify genes that require the interaction between ING2 and PtdIns(5)P for etoposide-mediated gene regulation

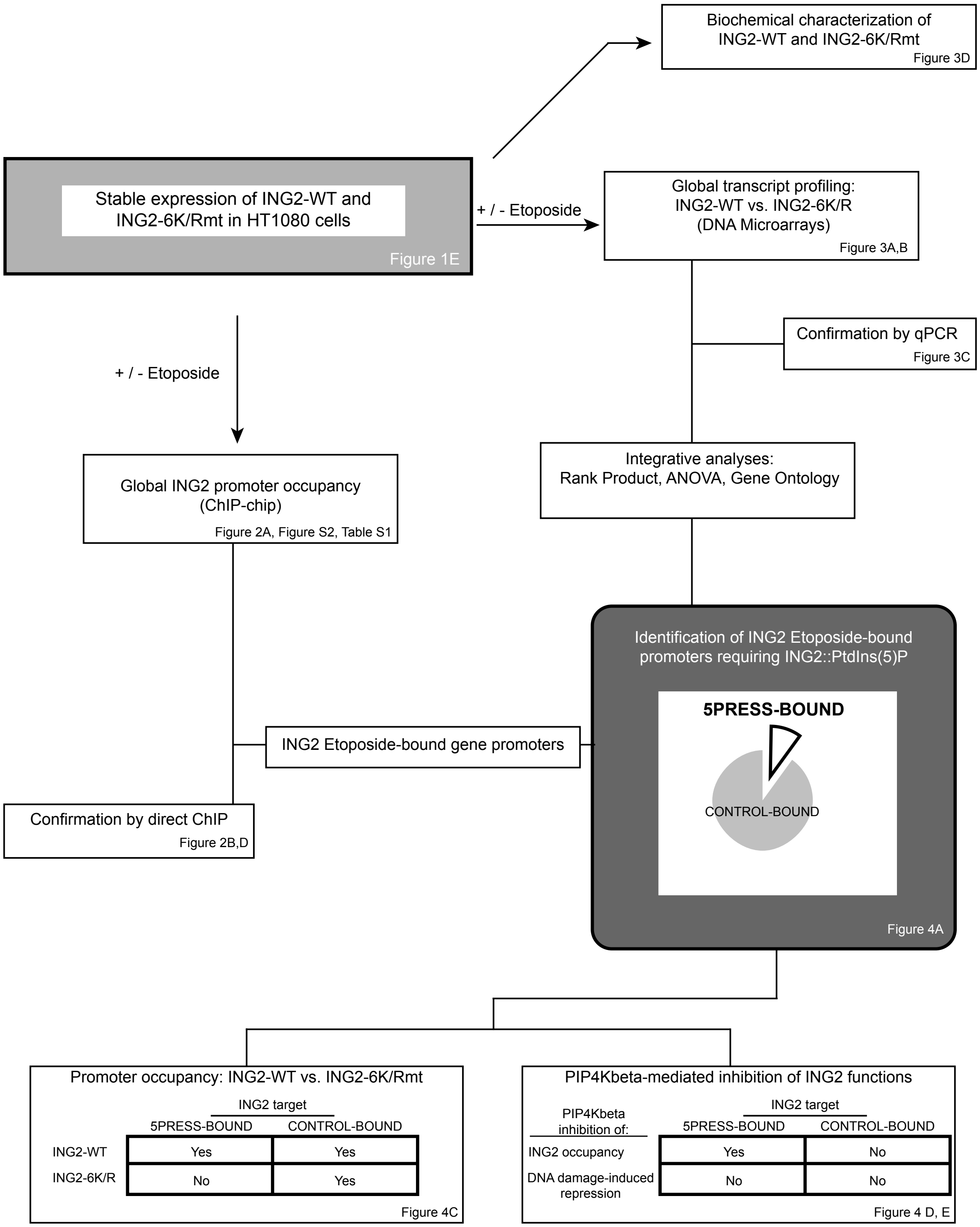

Supplementary Figure 2: ING2 distribution in the promoters of ING2 etoposide-bound genes. Heatmap displays of ING2 occupancy at ING2 ET-bound genes in the presence and absence of a 1-hour etoposide treatment (100microM); average enrichment calculatd every 250bp for each gene. Associated with Figure 2A.

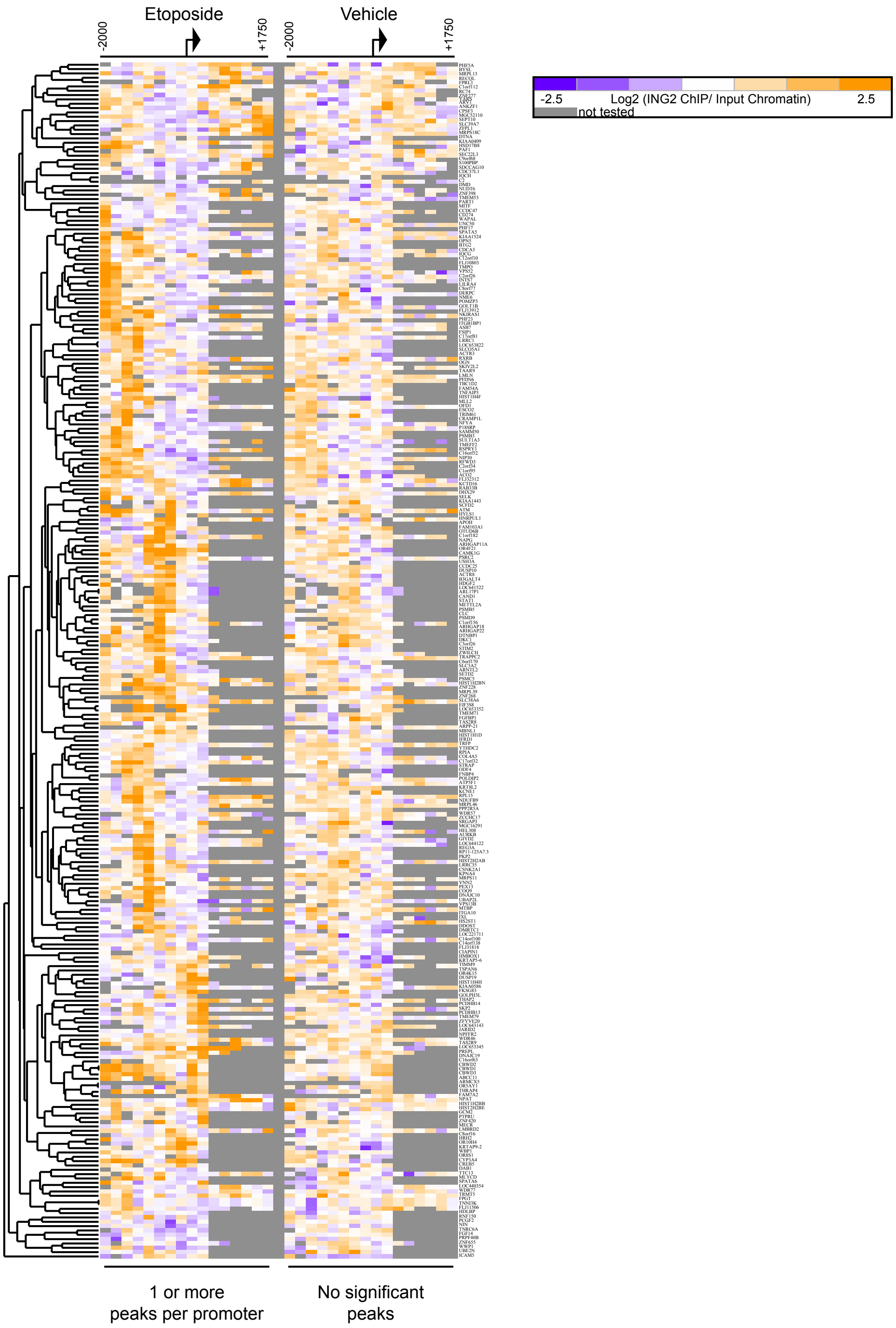

Supplementary Figure 3: Full scans of panel from Figure 1 A) Coomassie of GST-ING2 mutant panel from Figure 1A. Immunoblot analysis of pellets from peptide pull-down assays with B) ING2-WT (PHD-PBR), C) ING2-D230A, D) ING2-6K/Rmt, or E) ING2-D-6K/Rmt. Immunoblot analysis of pellets from liposome pull-down assays with F) ING2-WT (PHD-PBR), G) ING2-D230A, H) ING2-6K/Rmt, or I) ING2-D-6K/Rmt. Whole-cell extracts from HT1080 cell lines stably-expressing ING2 constructs probed with J) anti-ING2, K) anti-FLAG, or L) anti-tubulin

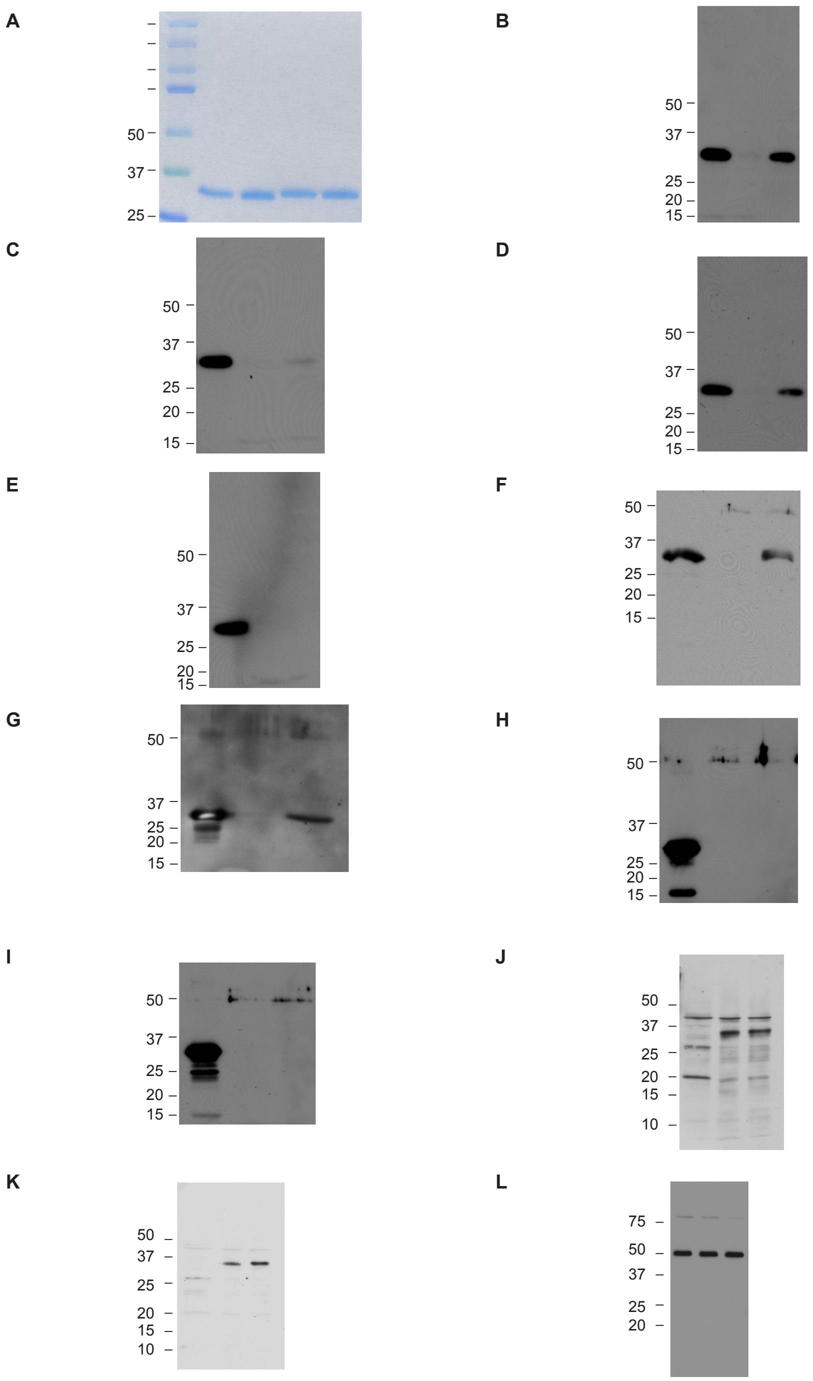

Supplementary Figure 4: Full scan of panels fromFigure 3D Immunoblot of FLAG-ING2 complexes probed with A) anti-FLAG, B) anti-SAP30, C) anti-HDAC1, or D) anti-SIN3A. Immunoblot of HDAC reactions in the absence and presence of trichostatin A (TSA): E,F) anti-H3, G,H) anti-H3K9Ac, or I,J) anti-H3K27Ac.

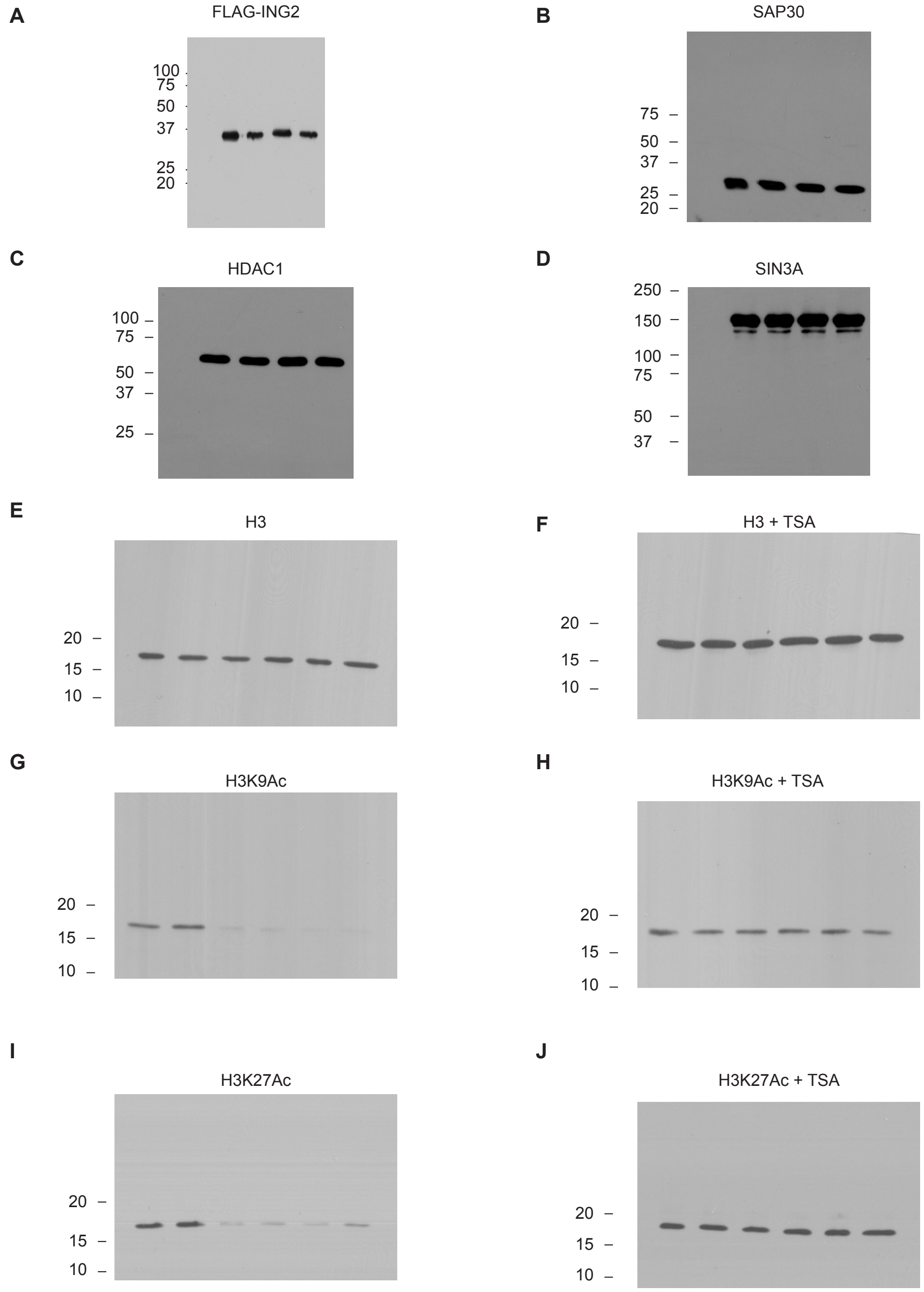

Supplementary Table 1: ING2 promoter distribution changes in response to etoposide. Peak Score = log2 (ING2 IP / Input chromatin) of the 4th largest probe in the ChIP-chip peak (see Methods). FDR = False Discovery Rate. Associated with Figure 2.

Gene ID	V-01		V-02		ET-01		ET-02		ING2 ET-BOUND
	Peak Score	FDR	Peak Score	FDR	Peak Score	FDR	Peak Score	FDR	
ACACA	0.72	9.9E-02	N/A	N/A	N/A	N/A	N/A	N/A	No
ACBD5	0.61	1.9E-01	N/A	N/A	N/A	N/A	N/A	N/A	No
ADNP	0.84	1.5E-01	N/A	N/A	N/A	N/A	N/A	N/A	No
AFF3	0.61	1.9E-01	N/A	N/A	N/A	N/A	N/A	N/A	No
AGPAT5	1.01	9.5E-02	N/A	N/A	N/A	N/A	N/A	N/A	No
ALKBH8	1.25	1.8E-02	N/A	N/A	1.38	1.2E-02	1.48	3.8E-03	No
APXL2	1.18	2.1E-02	N/A	N/A	N/A	N/A	N/A	N/A	No
ARHGDIG	0.98	1.4E-01	N/A	N/A	N/A	N/A	N/A	N/A	No
AXIN1	1.01	0.0E+00	N/A	N/A	N/A	N/A	N/A	N/A	No
BDH1	0.99	9.5E-02	N/A	N/A	N/A	N/A	N/A	N/A	No
BM88	0.84	1.5E-01	N/A	N/A	N/A	N/A	N/A	N/A	No
C17orf49	0.98	1.4E-01	N/A	N/A	N/A	N/A	N/A	N/A	No
C1orf55	0.76	1.7E-01	N/A	N/A	N/A	N/A	N/A	N/A	No
C21orf84	0.84	1.5E-01	N/A	N/A	N/A	N/A	N/A	N/A	No
C9orf111	1.08	7.6E-02	N/A	N/A	N/A	N/A	N/A	N/A	No
CA5B	0.95	1.4E-01	N/A	N/A	N/A	N/A	N/A	N/A	No
CD7	0.65	1.8E-01	N/A	N/A	N/A	N/A	N/A	N/A	No
CLIPR-59	0.9	1.5E-01	N/A	N/A	N/A	N/A	N/A	N/A	No
COBRA1	0.88	1.5E-01	N/A	N/A	N/A	N/A	N/A	N/A	No
COMT	0.97	1.4E-01	N/A	N/A	N/A	N/A	N/A	N/A	No
CORO1B	0.74	1.9E-01	N/A	N/A	N/A	N/A	N/A	N/A	No
CREB3	1.11	6.1E-02	N/A	N/A	N/A	N/A	N/A	N/A	No
DNAJB9	0.82	5.3E-02	N/A	N/A	N/A	N/A	N/A	N/A	No
DNMT3L	0.84	7.0E-02	N/A	N/A	N/A	N/A	N/A	N/A	No
DOK1	0.8	1.6E-01	N/A	N/A	N/A	N/A	N/A	N/A	No
DTX1	0.74	2.6E-02	N/A	N/A	N/A	N/A	N/A	N/A	No
DYRK2	0.64	1.8E-01	N/A	N/A	N/A	N/A	N/A	N/A	No
ECGF1	1	9.5E-02	N/A	N/A	N/A	N/A	N/A	N/A	No

EDG4	1.1	7.6E-02	N/A	N/A	N/A	N/A	N/A	N/A	No
ELF4	0.85	1.5E-01	N/A	N/A	N/A	N/A	N/A	N/A	No
EPN2	0.75	1.9E-01	N/A	N/A	N/A	N/A	N/A	N/A	No
EYA3	0.86	1.5E-01	N/A	N/A	N/A	N/A	N/A	N/A	No
FLJ23191	0.79	1.6E-01	N/A	N/A	N/A	N/A	N/A	N/A	No
FLJ39237	0.78	1.7E-01	N/A	N/A	N/A	N/A	N/A	N/A	No
FLJ41484	1.03	8.1E-02	N/A	N/A	N/A	N/A	N/A	N/A	No
FLJ42842	0.62	1.9E-01	N/A	N/A	N/A	N/A	N/A	N/A	No
FOXO3A	0.73	1.9E-01	N/A	N/A	N/A	N/A	N/A	N/A	No
FRMD6	0.74	1.2E-01	N/A	N/A	N/A	N/A	N/A	N/A	No
GAB4	0.71	9.9E-02	N/A	N/A	N/A	N/A	N/A	N/A	No
GPR35	0.77	1.2E-01	N/A	N/A	N/A	N/A	N/A	N/A	No
GPR68	0.96	1.4E-01	N/A	N/A	N/A	N/A	N/A	N/A	No
GRHL1	0.77	1.7E-01	N/A	N/A	N/A	N/A	N/A	N/A	No
GSCL	0.84	1.5E-01	N/A	N/A	N/A	N/A	N/A	N/A	No
HGS	1.05	1.6E-01	N/A	N/A	N/A	N/A	N/A	N/A	No
HMG20B	0.8	1.7E-01	N/A	N/A	N/A	N/A	N/A	N/A	No
HMX2	0.78	1.7E-01	N/A	N/A	N/A	N/A	N/A	N/A	No
HOMER2	0.68	1.5E-01	N/A	N/A	N/A	N/A	N/A	N/A	No
IDI1	1.04	8.1E-02	N/A	N/A	N/A	N/A	N/A	N/A	No
IFT80	1.44	0.0E+00	N/A	N/A	2.11	1.0E-02	1.58	0.0E+00	No
IGF2	0.71	9.9E-02	N/A	N/A	N/A	N/A	N/A	N/A	No
IGF2AS	0.71	9.9E-02	N/A	N/A	N/A	N/A	N/A	N/A	No
ITGA2	0.99	9.5E-02	N/A	N/A	N/A	N/A	N/A	N/A	No
JMJD1B	0.86	1.5E-01	N/A	N/A	N/A	N/A	N/A	N/A	No
JUB	0.71	9.9E-02	N/A	N/A	N/A	N/A	N/A	N/A	No
KCNN4	1.03	9.5E-02	N/A	N/A	N/A	N/A	N/A	N/A	No
KCNS2	0.62	1.9E-01	N/A	N/A	N/A	N/A	N/A	N/A	No
KLK4	1.17	4.4E-02	N/A	N/A	N/A	N/A	N/A	N/A	No
LDB1	1.55	0.0E+00	N/A	N/A	N/A	N/A	N/A	N/A	No
LIME1	0.78	1.7E-01	N/A	N/A	N/A	N/A	N/A	N/A	No
LINS1	1.26	0.0E+00	N/A	N/A	2.36	0.0E+00	1.21	8.9E-03	No
LOC338328	1.07	7.6E-02	N/A	N/A	N/A	N/A	N/A	N/A	No
LOC401565	0.75	1.9E-01	N/A	N/A	N/A	N/A	N/A	N/A	No
LOC440836	1	9.5E-02	N/A	N/A	N/A	N/A	N/A	N/A	No

LOC441476	0.75	1.9E-01	N/A	N/A	N/A	N/A	N/A	N/A	No
LOC643986	0.85	1.5E-01	N/A	N/A	N/A	N/A	N/A	N/A	No
LOC644248	0.73	1.9E-01	N/A	N/A	N/A	N/A	N/A	N/A	No
LOC646716	1.23	2.1E-02	N/A	N/A	N/A	N/A	N/A	N/A	No
LOC647115	0.61	1.9E-01	N/A	N/A	N/A	N/A	N/A	N/A	No
LOC653802	1.02	8.1E-02	N/A	N/A	N/A	N/A	N/A	N/A	No
LOXL3	0.8	1.6E-01	N/A	N/A	N/A	N/A	N/A	N/A	No
LRRC8D	0.99	9.5E-02	N/A	N/A	N/A	N/A	N/A	N/A	No
MAN2A1	0.73	1.9E-01	N/A	N/A	N/A	N/A	N/A	N/A	No
MGC13024	0.72	5.3E-02	N/A	N/A	N/A	N/A	N/A	N/A	No
MGC2752	1.43	0.0E+00	N/A	N/A	N/A	N/A	N/A	N/A	No
MGC40574	0.86	5.3E-02	N/A	N/A	N/A	N/A	N/A	N/A	No
MRPL41	1.08	7.6E-02	N/A	N/A	N/A	N/A	N/A	N/A	No
MTERFD1	1.27	0.0E+00	N/A	N/A	N/A	N/A	N/A	N/A	No
MUCDHL	1.25	0.0E+00	N/A	N/A	N/A	N/A	N/A	N/A	No
MUM1	0.63	1.9E-01	N/A	N/A	N/A	N/A	N/A	N/A	No
NDUFA7	0.63	1.9E-01	N/A	N/A	N/A	N/A	N/A	N/A	No
NRXN2	0.8	1.6E-01	N/A	N/A	N/A	N/A	N/A	N/A	No
NT5DC3	0.74	1.9E-01	N/A	N/A	N/A	N/A	N/A	N/A	No
OGFR	0.81	1.6E-01	N/A	N/A	N/A	N/A	N/A	N/A	No
PANK2	0.62	1.9E-01	N/A	N/A	N/A	N/A	N/A	N/A	No
PDIA2	0.98	1.4E-01	N/A	N/A	N/A	N/A	N/A	N/A	No
PHYHIP	0.75	1.9E-01	N/A	N/A	N/A	N/A	N/A	N/A	No
PLXNA1	0.99	9.5E-02	N/A	N/A	N/A	N/A	N/A	N/A	No
POU4F2	0.95	1.4E-01	N/A	N/A	N/A	N/A	N/A	N/A	No
PRRX2	0.82	1.6E-01	N/A	N/A	N/A	N/A	N/A	N/A	No
PTDSS1	1.27	0.0E+00	N/A	N/A	N/A	N/A	N/A	N/A	No
RAB3B	0.73	1.9E-01	N/A	N/A	N/A	N/A	N/A	N/A	No
RAB40C	0.82	1.6E-01	N/A	N/A	N/A	N/A	N/A	N/A	No
RALGDS	0.75	1.2E-01	N/A	N/A	N/A	N/A	N/A	N/A	No
REL	0.83	7.0E-02	N/A	N/A	N/A	N/A	N/A	N/A	No
RGS11	0.98	1.4E-01	N/A	N/A	N/A	N/A	N/A	N/A	No
RIMS3	0.73	1.9E-01	N/A	N/A	N/A	N/A	N/A	N/A	No
RP13-360B22.2	0.7	9.9E-02	N/A	N/A	N/A	N/A	N/A	N/A	No
RPS28	0.63	1.9E-01	N/A	N/A	N/A	N/A	N/A	N/A	No

RYBP	0.7	9.9E-02	N/A	N/A	N/A	N/A	N/A	N/A	No
SCT	1.25	0.0E+00	N/A	N/A	N/A	N/A	N/A	N/A	No
SETMAR	0.77	1.7E-01	N/A	N/A	N/A	N/A	N/A	N/A	No
SF3B14	1.02	8.1E-02	N/A	N/A	N/A	N/A	N/A	N/A	No
SLC12A5	0.75	1.9E-01	N/A	N/A	N/A	N/A	N/A	N/A	No
SLC16A3	0.78	1.7E-01	N/A	N/A	N/A	N/A	N/A	N/A	No
SLC17A4	0.8	1.6E-01	N/A	N/A	N/A	N/A	N/A	N/A	No
SLC25A1	0.71	9.9E-02	N/A	N/A	N/A	N/A	N/A	N/A	No
SLC2A4RG	0.78	1.7E-01	N/A	N/A	N/A	N/A	N/A	N/A	No
SMAD2	0.79	1.6E-01	N/A	N/A	N/A	N/A	N/A	N/A	No
SMC4L1	1.44	0.0E+00	N/A	N/A	2.11	1.0E-02	1.58	0.0E+00	No
SNCA	0.73	1.9E-01	N/A	N/A	N/A	N/A	N/A	N/A	No
SNN	1.34	0.0E+00	N/A	N/A	N/A	N/A	N/A	N/A	No
SPHK1	0.75	1.2E-01	N/A	N/A	N/A	N/A	N/A	N/A	No
SYTL4	0.95	1.4E-01	N/A	N/A	N/A	N/A	N/A	N/A	No
TADA2L	0.72	9.9E-02	N/A	N/A	N/A	N/A	N/A	N/A	No
TCEB2	0.79	1.7E-01	N/A	N/A	N/A	N/A	N/A	N/A	No
TDRD7	0.62	1.9E-01	N/A	N/A	N/A	N/A	N/A	N/A	No
THAP5	0.82	5.3E-02	N/A	N/A	N/A	N/A	N/A	N/A	No
TMEM121	0.8	5.3E-02	N/A	N/A	N/A	N/A	N/A	N/A	No
TRERF1	1.02	8.1E-02	N/A	N/A	N/A	N/A	N/A	N/A	No
TRIM42	0.83	1.5E-01	N/A	N/A	N/A	N/A	N/A	N/A	No
TUB	0.74	1.9E-01	N/A	N/A	N/A	N/A	N/A	N/A	No
TXNRD2	0.97	1.4E-01	N/A	N/A	N/A	N/A	N/A	N/A	No
UGP2	0.61	1.9E-01	N/A	N/A	N/A	N/A	N/A	N/A	No
UNQ830	0.61	1.9E-01	N/A	N/A	N/A	N/A	N/A	N/A	No
USP21	0.99	1.9E-01	N/A	N/A	N/A	N/A	N/A	N/A	No
VGF	0.69	5.3E-02	N/A	N/A	N/A	N/A	N/A	N/A	No
WDR26	0.73	1.9E-01	N/A	N/A	N/A	N/A	N/A	N/A	No
WDR37	1.04	8.1E-02	N/A	N/A	N/A	N/A	N/A	N/A	No
WHSC1	1.08	5.9E-02	N/A	N/A	3.81	0.0E+00	2.16	0.0E+00	No
ZBTB1	0.61	1.9E-01	N/A	N/A	N/A	N/A	N/A	N/A	No
ZBTB25	0.61	1.9E-01	N/A	N/A	N/A	N/A	N/A	N/A	No
ZFP36L2	0.86	5.3E-02	N/A	N/A	N/A	N/A	N/A	N/A	No
ZFP90	0.75	1.9E-01	N/A	N/A	N/A	N/A	N/A	N/A	No

ZNF131	0.7	9.9E-02	N/A	N/A	N/A	N/A	N/A	N/A	No
ZNF42	1.43	0.0E+00	N/A	N/A	N/A	N/A	N/A	N/A	No
ZNF583	1.23	2.1E-02	N/A	N/A	N/A	N/A	N/A	N/A	No
C1orf152	N/A	N/A	0.8	2.0E-01	N/A	N/A	N/A	N/A	No
CASP14	N/A	N/A	1.55	0.0E+00	N/A	N/A	N/A	N/A	No
DEFB123	N/A	N/A	0.81	2.0E-01	N/A	N/A	N/A	N/A	No
DPCR1	N/A	N/A	1.24	0.0E+00	N/A	N/A	N/A	N/A	No
DUB3	N/A	N/A	0.93	3.5E-02	N/A	N/A	N/A	N/A	No
FBXW4	N/A	N/A	0.87	1.4E-01	N/A	N/A	N/A	N/A	No
FLJ20920	N/A	N/A	1.46	3.5E-02	N/A	N/A	N/A	N/A	No
FLJ30934	N/A	N/A	0.97	0.0E+00	N/A	N/A	N/A	N/A	No
FRG1	N/A	N/A	0.8	2.0E-01	N/A	N/A	N/A	N/A	No
KAAG1	N/A	N/A	1.74	0.0E+00	N/A	N/A	N/A	N/A	No
NBPF20	N/A	N/A	0.8	2.0E-01	N/A	N/A	N/A	N/A	No
NBPF8	N/A	N/A	0.8	2.0E-01	N/A	N/A	N/A	N/A	No
NIPSNAP3A	N/A	N/A	0.86	1.4E-01	N/A	N/A	N/A	N/A	No
OR4F29	N/A	N/A	0.8	2.0E-01	2.83	0.0E+00	0.8	5.5E-02	No
OR4F3	N/A	N/A	0.8	2.0E-01	2.88	0.0E+00	0.8	5.5E-02	No
ZNF572	N/A	N/A	1.04	1.1E-01	N/A	N/A	N/A	N/A	No
ABCC11	N/A	N/A	N/A	N/A	1.46	3.1E-03	1.52	0.0E+00	Yes
ACO2	N/A	N/A	N/A	N/A	1.71	1.2E-02	1.55	0.0E+00	Yes
ACTR3	N/A	N/A	N/A	N/A	1.13	1.0E-01	0.71	1.6E-01	Yes
ACTR8	N/A	N/A	N/A	N/A	1.88	2.1E-02	1.03	6.2E-02	Yes
ANKZF1	N/A	N/A	N/A	N/A	2	2.8E-02	1.5	0.0E+00	Yes
APOH	N/A	N/A	N/A	N/A	1.69	2.5E-02	0.71	1.6E-01	Yes
ARHGAP11A	N/A	N/A	N/A	N/A	2.59	4.5E-04	0.71	1.6E-01	Yes
ARHGAP18	N/A	N/A	N/A	N/A	1.53	4.6E-02	0.84	5.7E-02	Yes
ARHGAP22	N/A	N/A	N/A	N/A	1.6	2.9E-02	0.86	1.4E-01	Yes
ARL17P1	N/A	N/A	N/A	N/A	1.9	2.9E-02	1.21	8.9E-03	Yes
ARMCX5	N/A	N/A	N/A	N/A	1.18	7.4E-02	0.7	1.6E-01	Yes
ARNTL2	N/A	N/A	N/A	N/A	2.38	1.1E-03	1.33	4.4E-03	Yes
ARPP-21	N/A	N/A	N/A	N/A	1.2	1.3E-01	0.94	1.1E-01	Yes
ARV1	N/A	N/A	N/A	N/A	1.2	1.1E-01	0.84	1.8E-01	Yes
ASB7	N/A	N/A	N/A	N/A	1.69	3.4E-02	1.21	8.9E-03	Yes
ATM	N/A	N/A	N/A	N/A	1.86	9.5E-03	1.4	2.4E-03	Yes

ATP5F1	N/A	N/A	N/A	N/A	1.09	1.2E-01	0.92	1.1E-01	Yes
AURKB	N/A	N/A	N/A	N/A	2.38	7.5E-03	1.12	2.3E-02	Yes
B3GALT4	N/A	N/A	N/A	N/A	2.02	2.8E-02	0.88	1.4E-01	Yes
BTG2	N/A	N/A	N/A	N/A	1.41	6.2E-02	1.17	1.7E-02	Yes
BYSL	N/A	N/A	N/A	N/A	0.98	1.9E-01	1.22	0.0E+00	Yes
C12orf10	N/A	N/A	N/A	N/A	1.38	6.2E-02	0.98	1.7E-01	Yes
C14orf100	N/A	N/A	N/A	N/A	1	1.7E-01	0.8	9.6E-02	Yes
C14orf138	N/A	N/A	N/A	N/A	1.31	1.0E-01	0.85	5.7E-02	Yes
C16orf52	N/A	N/A	N/A	N/A	1.66	1.6E-02	0.82	1.8E-01	Yes
C16orf63	N/A	N/A	N/A	N/A	0.99	1.7E-01	0.82	1.8E-01	Yes
C17orf32	N/A	N/A	N/A	N/A	2.69	0.0E+00	1	6.2E-02	Yes
C17orf81	N/A	N/A	N/A	N/A	1.21	1.7E-02	1.37	2.4E-03	Yes
C1orf112	N/A	N/A	N/A	N/A	1.14	1.5E-01	1.26	3.8E-02	Yes
C1orf156	N/A	N/A	N/A	N/A	1.14	1.5E-01	1.26	3.8E-02	Yes
C1orf182	N/A	N/A	N/A	N/A	1.2	9.0E-02	0.92	1.3E-02	Yes
C1orf95	N/A	N/A	N/A	N/A	1.14	1.3E-01	0.84	5.7E-02	Yes
C2	N/A	N/A	N/A	N/A	1.96	1.2E-02	1.05	4.3E-02	Yes
C2orf26	N/A	N/A	N/A	N/A	1.78	1.2E-02	0.67	1.9E-01	Yes
C2orf34	N/A	N/A	N/A	N/A	1.35	1.6E-02	1.25	7.5E-03	Yes
C3orf26	N/A	N/A	N/A	N/A	1.26	1.1E-01	1.11	2.9E-02	Yes
C6orf170	N/A	N/A	N/A	N/A	0.98	7.5E-02	1.77	0.0E+00	Yes
C8orf16	N/A	N/A	N/A	N/A	1.08	1.5E-01	0.87	4.4E-02	Yes
C8orf77	N/A	N/A	N/A	N/A	1.51	4.6E-02	1.25	7.5E-03	Yes
C9orf68	N/A	N/A	N/A	N/A	1.14	1.3E-01	0.84	1.8E-01	Yes
CAMK1G	N/A	N/A	N/A	N/A	2.28	3.1E-03	1.09	2.9E-02	Yes
CAND1	N/A	N/A	N/A	N/A	1.59	3.4E-02	0.68	1.3E-01	Yes
CBWD1	N/A	N/A	N/A	N/A	2.72	0.0E+00	1.46	1.3E-02	Yes
CBWD2	N/A	N/A	N/A	N/A	2.27	3.1E-03	1.46	1.3E-02	Yes
CBWD3	N/A	N/A	N/A	N/A	2.61	6.2E-04	1.42	3.1E-03	Yes
CCDC25	N/A	N/A	N/A	N/A	1.35	7.4E-02	1.33	4.0E-03	Yes
CCDC47	N/A	N/A	N/A	N/A	1.8	1.9E-02	0.71	1.6E-01	Yes
CD274	N/A	N/A	N/A	N/A	1.04	1.7E-01	0.96	1.7E-01	Yes
CDC37L1	N/A	N/A	N/A	N/A	1.14	1.3E-01	0.84	1.8E-01	Yes
CDCA5	N/A	N/A	N/A	N/A	2.07	2.0E-02	1.06	4.3E-02	Yes
CIAPIN1	N/A	N/A	N/A	N/A	2.65	0.0E+00	0.7	1.6E-01	Yes

CLC	N/A	N/A	N/A	N/A	1.26	9.8E-02	0.85	1.8E-01	Yes
CLYBL	N/A	N/A	N/A	N/A	1.29	1.0E-01	0.7	1.6E-01	Yes
COL4A5	N/A	N/A	N/A	N/A	0.99	1.0E-01	0.82	5.7E-02	Yes
COL4A6	N/A	N/A	N/A	N/A	0.99	1.0E-01	0.82	5.7E-02	Yes
COQ9	N/A	N/A	N/A	N/A	2.65	0.0E+00	0.7	1.6E-01	Yes
CPSF3	N/A	N/A	N/A	N/A	1.46	6.0E-03	0.87	2.1E-02	Yes
CRAMP1L	N/A	N/A	N/A	N/A	1.82	1.6E-02	0.99	6.2E-02	Yes
CREB5	N/A	N/A	N/A	N/A	1.71	2.9E-02	0.72	1.6E-01	Yes
CSNK2A1	N/A	N/A	N/A	N/A	1.34	4.1E-02	0.91	1.1E-01	Yes
CXorf50	N/A	N/A	N/A	N/A	1.08	9.8E-02	0.94	8.5E-02	Yes
CYP3A4	N/A	N/A	N/A	N/A	2.1	8.1E-03	1.22	8.9E-03	Yes
DAB1	N/A	N/A	N/A	N/A	2.07	1.9E-03	0.88	4.4E-02	Yes
DDOST	N/A	N/A	N/A	N/A	2.01	1.0E-02	1.72	0.0E+00	Yes
DERPC	N/A	N/A	N/A	N/A	1.09	1.3E-01	0.66	1.9E-01	Yes
DHX29	N/A	N/A	N/A	N/A	1.77	2.1E-02	0.88	2.1E-02	Yes
DKC1	N/A	N/A	N/A	N/A	1.32	4.6E-02	0.74	1.2E-01	Yes
DMD	N/A	N/A	N/A	N/A	1.36	4.1E-02	0.9	1.1E-01	Yes
DMRTC1	N/A	N/A	N/A	N/A	1.08	9.8E-02	0.94	8.5E-02	Yes
DNAJC10	N/A	N/A	N/A	N/A	1.35	7.4E-02	1.12	7.7E-02	Yes
DNAJC19	N/A	N/A	N/A	N/A	1.66	4.1E-02	0.9	1.4E-01	Yes
DTNA	N/A	N/A	N/A	N/A	1.24	9.8E-02	0.78	9.6E-02	Yes
DTNBP1	N/A	N/A	N/A	N/A	1.42	6.2E-02	1.13	2.3E-02	Yes
DUSP10	N/A	N/A	N/A	N/A	1.2	1.1E-01	1.05	5.5E-03	Yes
DUSP19	N/A	N/A	N/A	N/A	1.95	1.2E-02	0.75	1.2E-01	Yes
EIF3S8	N/A	N/A	N/A	N/A	2.29	9.8E-02	1.15	6.0E-02	Yes
ESCO2	N/A	N/A	N/A	N/A	1.35	7.4E-02	1.33	4.0E-03	Yes
FAM103A1	N/A	N/A	N/A	N/A	1.01	1.9E-01	1.71	0.0E+00	Yes
FAM54A	N/A	N/A	N/A	N/A	1.91	1.6E-02	0.92	1.1E-01	Yes
FAM7A2	N/A	N/A	N/A	N/A	2.3	4.7E-03	0.96	8.5E-02	Yes
FGF14	N/A	N/A	N/A	N/A	0.93	1.9E-01	0.82	1.8E-01	Yes
FGFBP1	N/A	N/A	N/A	N/A	1.29	1.8E-02	1.41	3.1E-03	Yes
FKSG83	N/A	N/A	N/A	N/A	2.13	6.7E-03	0.92	2.0E-01	Yes
FLJ10803	N/A	N/A	N/A	N/A	1.54	4.6E-02	0.72	1.6E-01	Yes
FLJ11506	N/A	N/A	N/A	N/A	1.01	1.9E-01	1.29	4.4E-03	Yes
FLJ13912	N/A	N/A	N/A	N/A	2.49	0.0E+00	0.95	1.8E-02	Yes

FLJ31818	N/A	N/A	N/A	N/A	0.99	1.9E-01	0.84	1.8E-01	Yes
FLJ32312	N/A	N/A	N/A	N/A	2	1.0E-02	1.04	4.3E-02	Yes
FNBP4	N/A	N/A	N/A	N/A	1.01	1.7E-01	0.89	1.4E-01	Yes
FPGT	N/A	N/A	N/A	N/A	1.14	1.3E-01	0.92	1.1E-01	Yes
FPRL1	N/A	N/A	N/A	N/A	1.69	2.9E-02	0.85	1.8E-01	Yes
FSIP1	N/A	N/A	N/A	N/A	1.29	9.8E-02	0.88	1.4E-01	Yes
GCM2	N/A	N/A	N/A	N/A	0.98	1.9E-01	0.88	1.4E-01	Yes
GIYD2	N/A	N/A	N/A	N/A	1.92	1.0E-02	0.86	1.4E-01	Yes
GOLPH3L	N/A	N/A	N/A	N/A	1.74	2.5E-02	0.88	4.4E-02	Yes
GOLT1B	N/A	N/A	N/A	N/A	1.91	1.2E-02	0.86	5.7E-02	Yes
HDGF2	N/A	N/A	N/A	N/A	1.69	2.9E-02	0.93	1.1E-01	Yes
HDLBP	N/A	N/A	N/A	N/A	2.27	3.1E-03	1.29	4.4E-03	Yes
HEL308	N/A	N/A	N/A	N/A	1.13	1.3E-01	1	6.9E-03	Yes
HIST1H1D	N/A	N/A	N/A	N/A	1.09	1.5E-01	1.43	1.8E-02	Yes
HIST1H2BB	N/A	N/A	N/A	N/A	1.2	1.1E-01	1.13	0.0E+00	Yes
HIST1H2BN	N/A	N/A	N/A	N/A	2.45	1.1E-03	0.92	2.0E-01	Yes
HIST1H4F	N/A	N/A	N/A	N/A	1.09	1.5E-01	1.43	1.8E-02	Yes
HIST1H4H	N/A	N/A	N/A	N/A	1.74	2.5E-02	0.84	1.8E-01	Yes
HIST2H2AB	N/A	N/A	N/A	N/A	1.2	1.1E-01	1.13	0.0E+00	Yes
HIST2H2BE	N/A	N/A	N/A	N/A	1.2	1.1E-01	1.13	0.0E+00	Yes
HMBOX1	N/A	N/A	N/A	N/A	1.02	1.7E-01	0.87	1.4E-01	Yes
HNRPUL1	N/A	N/A	N/A	N/A	2.29	3.1E-03	0.8	9.6E-02	Yes
HRH2	N/A	N/A	N/A	N/A	1.07	1.5E-01	0.83	1.8E-01	Yes
HS2ST1	N/A	N/A	N/A	N/A	1.25	9.8E-02	2.05	0.0E+00	Yes
HSD17B8	N/A	N/A	N/A	N/A	3.76	0.0E+00	0.92	1.1E-01	Yes
HYLS1	N/A	N/A	N/A	N/A	2.39	1.1E-03	0.89	1.4E-01	Yes
ICAM5	N/A	N/A	N/A	N/A	1.04	1.7E-01	0.97	1.7E-01	Yes
IFRD1	N/A	N/A	N/A	N/A	1.71	2.9E-02	0.72	1.6E-01	Yes
INTS7	N/A	N/A	N/A	N/A	1.63	3.4E-02	0.96	8.5E-02	Yes
IQCG	N/A	N/A	N/A	N/A	1.2	1.8E-02	0.94	3.1E-02	Yes
IQCH	N/A	N/A	N/A	N/A	1.01	1.9E-01	1.29	4.4E-03	Yes
ITGA10	N/A	N/A	N/A	N/A	0.98	1.9E-01	1.13	2.3E-02	Yes
ITGB1BP1	N/A	N/A	N/A	N/A	1.46	6.0E-03	0.87	2.1E-02	Yes
IXL	N/A	N/A	N/A	N/A	1.31	8.4E-02	0.93	1.1E-01	Yes
JARID2	N/A	N/A	N/A	N/A	1.42	8.8E-02	1.26	3.8E-02	Yes

KCNE1	N/A	N/A	N/A	N/A	1.75	2.1E-02	1.1	2.9E-02	Yes
KCTD16	N/A	N/A	N/A	N/A	1.29	8.4E-02	0.88	1.4E-01	Yes
KIAA0409	N/A	N/A	N/A	N/A	1.49	4.6E-02	1.1	2.9E-02	Yes
KIAA0586	N/A	N/A	N/A	N/A	1.47	4.6E-02	1.02	6.2E-02	Yes
KIAA1443	N/A	N/A	N/A	N/A	1.05	1.5E-01	1.02	6.2E-02	Yes
KIAA1524	N/A	N/A	N/A	N/A	2.34	4.7E-03	0.73	1.6E-01	Yes
KPNA4	N/A	N/A	N/A	N/A	1.43	7.4E-02	0.9	0.0E+00	Yes
KRT8L2	N/A	N/A	N/A	N/A	1.43	7.4E-02	0.9	0.0E+00	Yes
KRTAP5-6	N/A	N/A	N/A	N/A	1.17	1.1E-01	1.27	7.5E-03	Yes
KRTAP9-2	N/A	N/A	N/A	N/A	1.48	4.6E-02	0.71	1.6E-01	Yes
LILRA4	N/A	N/A	N/A	N/A	1.58	4.1E-02	1.01	1.5E-01	Yes
LMBRD2	N/A	N/A	N/A	N/A	1.24	9.8E-02	0.96	8.5E-02	Yes
LMLN	N/A	N/A	N/A	N/A	1.2	1.8E-02	0.94	3.1E-02	Yes
LOC221711	N/A	N/A	N/A	N/A	0.98	1.9E-01	0.88	1.4E-01	Yes
LOC440354	N/A	N/A	N/A	N/A	1.92	1.0E-02	0.86	1.4E-01	Yes
LOC641522	N/A	N/A	N/A	N/A	1.9	2.9E-02	1.21	8.9E-03	Yes
LOC643143	N/A	N/A	N/A	N/A	1.36	7.4E-02	1.05	5.5E-03	Yes
LOC644122	N/A	N/A	N/A	N/A	1.56	3.4E-02	0.86	1.4E-01	Yes
LOC653345	N/A	N/A	N/A	N/A	2.88	0.0E+00	0.71	1.6E-01	Yes
LOC653352	N/A	N/A	N/A	N/A	2.29	9.8E-02	1.15	6.0E-02	Yes
LOC653822	N/A	N/A	N/A	N/A	0.98	1.9E-01	0.71	1.6E-01	Yes
LRRC1	N/A	N/A	N/A	N/A	0.98	1.9E-01	0.71	1.6E-01	Yes
LRRC35	N/A	N/A	N/A	N/A	1.12	1.3E-01	0.89	1.4E-01	Yes
MAGEH1	N/A	N/A	N/A	N/A	1.27	5.4E-02	0.86	1.4E-01	Yes
MBNL1	N/A	N/A	N/A	N/A	2.4	3.1E-03	1.03	6.2E-02	Yes
MECR	N/A	N/A	N/A	N/A	1.47	8.3E-02	0.84	1.8E-01	Yes
METTL2A	N/A	N/A	N/A	N/A	1.21	9.8E-02	0.71	1.6E-01	Yes
MGC16291	N/A	N/A	N/A	N/A	1.65	2.5E-02	1.07	0.0E+00	Yes
MGC52110	N/A	N/A	N/A	N/A	1.03	1.4E-01	0.87	1.4E-01	Yes
MITF	N/A	N/A	N/A	N/A	1.94	1.9E-02	0.98	8.5E-02	Yes
MLL2	N/A	N/A	N/A	N/A	1.01	1.7E-01	1.2	1.7E-02	Yes
MLYCD	N/A	N/A	N/A	N/A	1.82	1.6E-02	0.82	1.8E-01	Yes
MRPL13	N/A	N/A	N/A	N/A	2.42	1.1E-03	0.79	0.0E+00	Yes
MRPL39	N/A	N/A	N/A	N/A	2.01	1.9E-03	0.85	5.7E-02	Yes
MRPL46	N/A	N/A	N/A	N/A	1.97	1.6E-02	0.79	9.6E-02	Yes

MRPS11	N/A	N/A	N/A	N/A	1.97	1.6E-02	0.79	9.6E-02	Yes
MRPS18C	N/A	N/A	N/A	N/A	4.08	0.0E+00	1	6.9E-03	Yes
MTBP	N/A	N/A	N/A	N/A	2.42	1.1E-03	0.79	0.0E+00	Yes
NAPG	N/A	N/A	N/A	N/A	2.48	4.5E-04	1.03	5.5E-03	Yes
NDUFB9	N/A	N/A	N/A	N/A	1.56	4.1E-02	0.96	8.5E-02	Yes
NFYA	N/A	N/A	N/A	N/A	1.85	1.9E-02	0.84	1.8E-01	Yes
NIN	N/A	N/A	N/A	N/A	1.89	1.2E-02	0.89	1.4E-01	Yes
NIP30	N/A	N/A	N/A	N/A	1.35	6.2E-02	1.32	4.0E-03	Yes
NKIRAS1	N/A	N/A	N/A	N/A	2.17	8.1E-03	0.94	1.1E-01	Yes
NME6	N/A	N/A	N/A	N/A	1.49	6.2E-02	1.11	8.7E-02	Yes
NPAT	N/A	N/A	N/A	N/A	1.86	9.5E-03	1.4	2.4E-03	Yes
NPFFR2	N/A	N/A	N/A	N/A	1.4	6.2E-02	0.83	5.7E-02	Yes
NUDT6	N/A	N/A	N/A	N/A	0.97	1.9E-01	0.87	1.4E-01	Yes
ODF4	N/A	N/A	N/A	N/A	1.69	2.5E-02	0.83	1.8E-01	Yes
OFD1	N/A	N/A	N/A	N/A	0.99	1.0E-01	1.06	2.9E-02	Yes
OGN	N/A	N/A	N/A	N/A	1.14	1.3E-01	0.75	1.2E-01	Yes
OPN5	N/A	N/A	N/A	N/A	1.14	3.8E-02	0.84	1.8E-01	Yes
OR10H4	N/A	N/A	N/A	N/A	2.4	1.6E-03	0.85	1.8E-01	Yes
OR4F21	N/A	N/A	N/A	N/A	2.69	0.0E+00	0.96	8.5E-02	Yes
OR4K15	N/A	N/A	N/A	N/A	1.73	2.1E-02	0.89	1.4E-01	Yes
OR5AY1	N/A	N/A	N/A	N/A	0.98	1.7E-01	0.88	1.4E-01	Yes
OR8S1	N/A	N/A	N/A	N/A	1.06	1.5E-01	0.86	0.0E+00	Yes
OTUD6B	N/A	N/A	N/A	N/A	1.08	1.5E-01	1	6.2E-02	Yes
P18SRP	N/A	N/A	N/A	N/A	0.97	1.9E-01	0.75	1.2E-01	Yes
PAF1	N/A	N/A	N/A	N/A	1.31	8.4E-02	0.93	1.1E-01	Yes
PART1	N/A	N/A	N/A	N/A	1.18	1.1E-01	1.33	4.0E-03	Yes
PCDHB13	N/A	N/A	N/A	N/A	1.29	8.4E-02	0.71	1.6E-01	Yes
PCDHB14	N/A	N/A	N/A	N/A	2.2	1.4E-02	0.88	1.4E-01	Yes
PCGF2	N/A	N/A	N/A	N/A	1.06	1.5E-01	0.96	8.5E-02	Yes
PDE4DIP	N/A	N/A	N/A	N/A	1.79	0.0E+00	0.84	5.7E-02	Yes
PEX13	N/A	N/A	N/A	N/A	2	1.0E-02	1.04	4.3E-02	Yes
PFDN6	N/A	N/A	N/A	N/A	0.82	1.4E-01	1.26	6.3E-03	Yes
PHF17	N/A	N/A	N/A	N/A	1.61	3.4E-02	0.92	3.1E-02	Yes
PHF23	N/A	N/A	N/A	N/A	1.21	1.7E-02	1.37	2.4E-03	Yes
PHF5A	N/A	N/A	N/A	N/A	1.71	1.2E-02	1.55	0.0E+00	Yes

PKP2	N/A	N/A	N/A	N/A	2.28	1.7E-03	1.07	4.3E-02	Yes
POLDIP2	N/A	N/A	N/A	N/A	2.69	0.0E+00	1	6.2E-02	Yes
POMZP3	N/A	N/A	N/A	N/A	2.48	7.5E-03	1.31	4.4E-03	Yes
PPP2R5A	N/A	N/A	N/A	N/A	1.36	7.4E-02	1.05	5.5E-03	Yes
PREPL	N/A	N/A	N/A	N/A	1.35	1.6E-02	1.25	7.5E-03	Yes
PRPF40B	N/A	N/A	N/A	N/A	1.43	5.4E-02	1.2	1.7E-02	Yes
PSMB3	N/A	N/A	N/A	N/A	1.06	1.5E-01	0.96	8.5E-02	Yes
PSMB5	N/A	N/A	N/A	N/A	2	8.1E-03	0.89	4.4E-02	Yes
PSMC5	N/A	N/A	N/A	N/A	1.58	3.4E-02	0.96	1.8E-02	Yes
PSMD9	N/A	N/A	N/A	N/A	1.22	9.8E-02	1.03	6.2E-02	Yes
PSRC2	N/A	N/A	N/A	N/A	1.8	1.9E-02	0.86	1.8E-01	Yes
PTPRU	N/A	N/A	N/A	N/A	1.47	8.3E-02	0.84	1.8E-01	Yes
RAB33B	N/A	N/A	N/A	N/A	1.61	3.4E-02	0.92	1.1E-01	Yes
RC74	N/A	N/A	N/A	N/A	1.02	1.7E-01	0.87	1.4E-01	Yes
RECQL	N/A	N/A	N/A	N/A	1.91	1.2E-02	0.86	5.7E-02	Yes
REG3A	N/A	N/A	N/A	N/A	1.19	1.3E-01	0.83	1.8E-01	Yes
RFWD3	N/A	N/A	N/A	N/A	1.77	1.0E-02	1.07	7.0E-03	Yes
RNF150	N/A	N/A	N/A	N/A	1.07	1.2E-01	0.83	1.8E-01	Yes
RP11-125A7.3	N/A	N/A	N/A	N/A	1.86	1.2E-02	1.23	6.3E-03	Yes
RPIA	N/A	N/A	N/A	N/A	1.19	1.1E-01	0.96	8.5E-02	Yes
RPL15	N/A	N/A	N/A	N/A	2.17	8.1E-03	0.94	1.1E-01	Yes
RSPRY1	N/A	N/A	N/A	N/A	1.09	3.8E-02	1.03	4.3E-02	Yes
RXRB	N/A	N/A	N/A	N/A	3.76	0.0E+00	0.92	1.1E-01	Yes
S100PBP	N/A	N/A	N/A	N/A	1.2	3.4E-02	0.92	1.1E-01	Yes
SAMM50	N/A	N/A	N/A	N/A	0.93	1.9E-01	0.84	5.7E-02	Yes
SCFD2	N/A	N/A	N/A	N/A	0.97	1.7E-01	1	6.9E-03	Yes
SDCCAG10	N/A	N/A	N/A	N/A	0.97	1.9E-01	0.75	1.2E-01	Yes
SEC22L3	N/A	N/A	N/A	N/A	1.03	1.9E-01	0.9	2.1E-02	Yes
SELK	N/A	N/A	N/A	N/A	1.6	3.2E-02	0.86	1.8E-01	Yes
SEPT10	N/A	N/A	N/A	N/A	0.97	1.9E-01	0.67	1.9E-01	Yes
SEPT15	N/A	N/A	N/A	N/A	1.25	9.8E-02	2.05	0.0E+00	Yes
SETD2	N/A	N/A	N/A	N/A	1.31	2.8E-02	0.73	1.6E-01	Yes
SKIV2L2	N/A	N/A	N/A	N/A	1.29	8.4E-02	0.88	2.1E-02	Yes
SKP2	N/A	N/A	N/A	N/A	1.24	9.8E-02	0.96	8.5E-02	Yes
SLC38A6	N/A	N/A	N/A	N/A	1.1	3.8E-02	0.76	1.2E-01	Yes

SLC39A7	N/A	N/A	N/A	N/A	1.14	1.3E-01	0.92	1.1E-01	Yes
SLC3A2	N/A	N/A	N/A	N/A	1.54	4.1E-02	0.93	3.1E-02	Yes
SLCO5A1	N/A	N/A	N/A	N/A	2.05	8.1E-03	0.71	1.6E-01	Yes
SPATA5	N/A	N/A	N/A	N/A	0.97	1.9E-01	0.87	1.4E-01	Yes
SPATA6	N/A	N/A	N/A	N/A	1.41	6.2E-02	0.84	1.8E-01	Yes
SRGAP3	N/A	N/A	N/A	N/A	1.54	5.4E-02	1.54	0.0E+00	Yes
STAT1	N/A	N/A	N/A	N/A	3.08	0.0E+00	1.12	2.3E-02	Yes
STIM2	N/A	N/A	N/A	N/A	1.56	2.9E-02	1	0.0E+00	Yes
STRAP	N/A	N/A	N/A	N/A	1.06	1.2E-01	0.81	9.6E-02	Yes
SULT1A3	N/A	N/A	N/A	N/A	1.92	1.0E-02	0.86	1.4E-01	Yes
TAAR9	N/A	N/A	N/A	N/A	1.53	4.6E-02	0.88	1.4E-01	Yes
TAS2R8	N/A	N/A	N/A	N/A	1.43	5.4E-02	0.98	8.5E-02	Yes
TAS2R9	N/A	N/A	N/A	N/A	1.43	5.4E-02	0.98	8.5E-02	Yes
TBC1D2	N/A	N/A	N/A	N/A	1.53	4.6E-02	1.38	2.4E-03	Yes
THAP2	N/A	N/A	N/A	N/A	1.8	1.9E-02	0.86	1.8E-01	Yes
THRAP4	N/A	N/A	N/A	N/A	2.38	9.8E-02	0.87	1.4E-01	Yes
TIMM9	N/A	N/A	N/A	N/A	1.47	4.6E-02	1.02	6.2E-02	Yes
TMEFF2	N/A	N/A	N/A	N/A	1.35	7.4E-02	0.92	3.1E-02	Yes
TMEM53	N/A	N/A	N/A	N/A	1.58	6.9E-02	1.01	1.5E-01	Yes
TMEM71	N/A	N/A	N/A	N/A	1.35	7.4E-02	0.87	1.4E-01	Yes
TMEM79	N/A	N/A	N/A	N/A	2.07	1.9E-03	1.05	4.3E-02	Yes
TMPO	N/A	N/A	N/A	N/A	3.02	0.0E+00	0.9	4.4E-02	Yes
TNFAIP3	N/A	N/A	N/A	N/A	0.98	1.9E-01	0.67	1.3E-01	Yes
TNNI3K	N/A	N/A	N/A	N/A	1.14	1.3E-01	0.92	1.1E-01	Yes
TNRC6A	N/A	N/A	N/A	N/A	2.08	6.0E-03	0.86	2.1E-02	Yes
TRAPPC2	N/A	N/A	N/A	N/A	0.99	1.0E-01	1.06	2.9E-02	Yes
TRFP	N/A	N/A	N/A	N/A	0.98	1.9E-01	1.22	0.0E+00	Yes
TRIM61	N/A	N/A	N/A	N/A	1.23	1.2E-01	1	1.5E-01	Yes
TRMT5	N/A	N/A	N/A	N/A	1.1	3.8E-02	0.76	1.2E-01	Yes
TSPAN6	N/A	N/A	N/A	N/A	0.94	1.5E-01	0.98	0.0E+00	Yes
TTC13	N/A	N/A	N/A	N/A	1.03	1.7E-01	0.84	1.8E-01	Yes
UBAP2L	N/A	N/A	N/A	N/A	1.9	1.6E-02	0.92	1.1E-01	Yes
UBE2N	N/A	N/A	N/A	N/A	1.01	1.7E-01	0.77	1.2E-01	Yes
UNC50	N/A	N/A	N/A	N/A	1.3	8.4E-02	0.87	1.4E-01	Yes
USH3A	N/A	N/A	N/A	N/A	1.2	1.3E-01	1.24	4.4E-02	Yes

VNN2	N/A	N/A	N/A	N/A	1.69	2.1E-02	0.92	1.1E-01	Yes
VPS13B	N/A	N/A	N/A	N/A	1.51	4.6E-02	0.83	1.8E-01	Yes
VPS52	N/A	N/A	N/A	N/A	2.02	2.8E-02	0.88	1.4E-01	Yes
WAPAL	N/A	N/A	N/A	N/A	1.24	1.1E-01	0.82	1.8E-01	Yes
WBP1	N/A	N/A	N/A	N/A	1.51	3.2E-02	0.92	1.1E-01	Yes
WDR46	N/A	N/A	N/A	N/A	0.82	1.4E-01	1.26	6.3E-03	Yes
WDR57	N/A	N/A	N/A	N/A	1.3	8.4E-02	0.84	1.8E-01	Yes
WDR77	N/A	N/A	N/A	N/A	1.09	1.2E-01	0.92	1.1E-01	Yes
WWP1	N/A	N/A	N/A	N/A	1.51	7.6E-02	1.08	2.9E-02	Yes
YARS	N/A	N/A	N/A	N/A	1.2	3.4E-02	0.92	1.1E-01	Yes
YTHDC2	N/A	N/A	N/A	N/A	1.13	1.3E-01	0.83	1.8E-01	Yes
ZCCHC17	N/A	N/A	N/A	N/A	1.3	8.4E-02	0.84	1.8E-01	Yes
ZFPL1	N/A	N/A	N/A	N/A	1.6	3.4E-02	1.06	4.3E-02	Yes
ZFYVE20	N/A	N/A	N/A	N/A	1.49	6.2E-02	1.2	6.0E-02	Yes
ZNF228	N/A	N/A	N/A	N/A	1.97	1.2E-02	1.23	5.8E-03	Yes
ZNF268	N/A	N/A	N/A	N/A	1.11	1.3E-01	0.86	5.7E-02	Yes
ZNF277	N/A	N/A	N/A	N/A	1.1	1.5E-01	0.89	1.4E-01	Yes
ZNF398	N/A	N/A	N/A	N/A	1.43	6.2E-02	1.27	3.8E-02	Yes
ZNF420	N/A	N/A	N/A	N/A	1.26	9.8E-02	0.72	1.6E-01	Yes
ZNF655	N/A	N/A	N/A	N/A	1.87	0.0E+00	0.97	0.0E+00	Yes
ZWILCH	N/A	N/A	N/A	N/A	1.29	9.8E-02	0.75	1.2E-01	Yes

Supplementary Table 2: Primers used in this study Probe# refers to probe in the Roche Universal Probe library.

ID	Assay	Forward (5' to 3')	Reverse (5' to 3')	Probe#	Associated Figure(s)
DKC1 (-1545)	ChIP	ctctttggagggaccgtct	gggaggtgatacctcagtga		1B
DKC1 (-1175)	ChIP	ggctgactccacttctgacc	gcactagcccgtgtgtgaatc		1B
DKC1 (-462)	ChIP	tggcacgcacactactccta	gaagctcggagaggttgaaa		1B
DKC1 (+380)	ChIP	aaagacatctgccgtgctg	tcgcatggagctgtaacg		1B
ACTA	ChIP	tctaaaaggggggatggaagc	gatgaactgccatgatgtgg		1D
AURKB	ChIP	gttagctgccgtgttgtttg	agcagaaaagaaggaagtgtgg		1D,E
PCFG2	ChIP	aacgcgggaaatctaggc	gatctgatcctcgcaatagctc		1D,E
GAPDH	Expression	agccacatcgctcagacac	gcccaatacgaccaaatcc	60	3C,4E
AURKB	Expression	attgctgacttcggctggt	gtccagggtgccacacat	69	3C
DKC1	Expression	gtggggtttaggtccaaagg	tttggcagactcatcctgct	87	3C
SKP2	Expression	ctgtctcaaggggtgattgc	ttcgataggtccatgtgctg	44	3C
UBE2N	Expression	cgcaggatcatcaaggaaa	aaataacgggcgttgctct	72	3C,4E
CIP2A	ChIP	ggatgccatgtggctagtgt	ccaagaccgtataggacctca	55	4C,D
UBE2N	ChIP	cgtgccttccaggaacttag	gcccttaacacattggattgtaa	54	4C,D
CIP2A	Expression	gaacagataagaaaagagttgagcatt	cgaccttctaattgtgcctttt	69	4E
